# Impact of the anti-aquaporin-4 autoantibody on inner retinal structure, function and structure-function associations in Japanese patients with optic neuritis

**DOI:** 10.1371/journal.pone.0171880

**Published:** 2017-02-15

**Authors:** Yoshiko Matsumoto, Sotaro Mori, Kaori Ueda, Takuji Kurimoto, Akiyasu Kanamori, Yuko Yamada, Ichiro Nakashima, Makoto Nakamura

**Affiliations:** 1 Department of Surgery, Division of Ophthalmology, Kobe University Graduate School of Medicine, Kobe, Japan; 2 Department of Neurology, Tohoku University School of Medicine, Sendai, Japan; Bascom Palmer Eye Institute, UNITED STATES

## Abstract

**Purpose:**

An autoantibody against aquaporin-4 (AQP4 Ab) is highly specific for neuromyelitis optica spectrum disorder and plays a pathogenic role in this disease. The purpose of this study was to investigate the impact of AQP4 Ab on inner retinal structure, function, and the structure−function relationships in eyes with optic neuritis.

**Methods:**

Thirty five eyes from 25 cases who had received visual function tests and RTVue optical coherence tomography (OCT) measurement at least six months after the latest episode of optic neuritis were enrolled. Patients with multiple sclerosis were excluded. AQP4 Ab was measured using a cell-based assay. Visual acuity, mean deviation (MD) of the Humphrey visual field SITA standard 30–2 tests, retinal nerve fiber layer (RNFL), ganglion cell complex (GCC) thicknesses, and other clinical variables were compared between the AQP4 Ab-positive and -negative groups. Parameters associated with visual functions were evaluated by generalized estimating equation (GEE) models.

**Results:**

The AQP4 Ab-positive group (20 eyes from 12 cases) had a higher proportion of bilateral involvement and longer duration of follow-up than the AQP4 Ab-negative group (15 eyes from 13 cases). Linear mixed effect models revealed worse MD and visual acuity in AQP4 Ab-positive eyes than those in AQP4 Ab-negative eyes after adjusting for within-patient inter-eye dependence, whereas there were no differences in RNFL and GCC thickness between the two groups. In seropositive eyes, GEE regression analyses revealed that depending on age and the number of recurrences of ON episodes, OCT parameters correlated strongly with MD and more weakly with visual acuity.

**Conclusions:**

Reductions in RNFL and GCC thickness were proportional to the visual field defect in eyes with AQP4 Ab but not in eyes without AQP4 Ab. The presence of AQP4 Ab probably plays a critical role in retinal ganglion cell loss in optic neuritis.

## Introduction

Neuromyelitis optica (NMO) is an autoimmune inflammatory disease of the central nervous system that preferably, but not exclusively, targets the optic nerve and spinal cord.[1] Serum autoantibodies against the water channel aquaporin-4 (AQP4 Ab) have been found to be highly specific for NMO and to play a pathogenic role in conjunction with complement activation.[1–4] The term NMO spectrum disorder (NMOSD) has been coined for conditions that do not meet all NMO diagnostic criteria but include AQP4 Ab-positive cases with first-attack longitudinally extensive transverse myelitis or recurrent (LETM) or bilateral optic neuritis (ON).[5] The latest version of NMO diagnostic criteria emphasized the presence of AQP4 Ab.[1] In other words, even patients with unilateral and first-attack ON are now defined as having NMOSD if they have AQP4 Ab.

Most previous studies have demonstrated that visual prognosis is worse if optic neuritis occurs in patients with NMOSD.[6–10] Even a single episode of ON is thought to be capable of causing legal blindness in about one-third of patients with NMOSD, and less than half of them show complete recovery of visual function.[6] An experimental study showed that exposure of rodent optic nerve to serum from AQP4 Ab-positive NMOSD patients led not only to the loss of optic nerve astrocytes, which abundantly express AQP4 but also to the death of retinal ganglion cells (RGCs), the essential neuronal components that transmit visual information from the retina to the brain.[11] Therefore, it is highly likely that AQP4 Ab has a pathological impact on the viability of human RGCs. Previous studies primarily compared retinal structure and function between NMOSD and either multiple sclerosis patients or controls based on clinical diagnostic criteria.[12–22] It has been found that 60%−80% of patients with definite NMO but only 5%−25% of patients with recurrent isolated ON were seropositive.[8] Therefore, the pathological significance of AQP4 Ab or AQP4 Ab-positive serum on retinal structural and functional outcomes is still unclear.

Optical coherence tomography (OCT) is a modality of near-infrared interferometry that can non-invasively analyze retinal layer structure. Recently introduced spectral-domain (SD) OCT has improved image acquisition speed and introduced an autosegmentation algorithm capable of measuring not only circumpapillary retinal nerve fiber layer (cpRNFL) thickness, the sole parameter measured by the older time-domain (TD) OCT, but also thickness of the ganglion cell complex (GCC) at the macula region, the layers (ganglion cell layer, inner plexiform layer, and macular RNFL) containing RGCs.[23] Although SD OCT evaluation of cpRNFL and inner macular retinal structures such as the GCC has been validated in studies of glaucomatous optic neuropathy,[24] numerous studies have also demonstrated that changes in cpRNFL and inner macular retinal structure are more severe in eyes with clinically diagnosed NMOSD compared to eyes with idiopathic ON or multiple sclerosis.[12–22, 25–30] However, these studies did not focus on the impact of AQP4 Ab presence on OCT parameter thinning. Indeed, in several studies, the proportion of seropositive patients with NMOSD was not described, [13,18–20,29] while in others [12,14,16,22,25,28] clinical parameters were not compared between AQP4 Ab-positive and -negative eyes within the NMOSD group (comprising 52% to 100% of AQP4 Ab-positive NMO patients in these studies). Few previous studies have evaluated the correlation of visual field damage with RNFL or GCC thickness changes in eyes with NMOSD.[16,27]

The purpose of this study was to elucidate the direct impact of AQP4 Ab presence on inner retinal structure, function, and structure−function relationships in ON by comparing OCT parameters and visual functions between patients with, and without, AQP4 Ab.

## Materials and methods

### Study design and ethics statement

This was a retrospective, cross-sectional study. The study protocol was approved by the institutional review board of Kobe University (UMIN 000006900 and 000006901), Japan, and adhered to the tenets of the Declaration of Helsinki. Written informed consent was obtained from each participant. We obtained consent from parents of patients under 20 years of age as well as assent from patients themselves under 20 years of age

### Subject selection and enrollment criteria

We reviewed the medical charts of 49 consecutive patients who had a history of ON in at least one eye and were treated by the Department of Ophthalmology, Kobe University Hospital, Japan, during the period 2006 to 2015. Diagnosis of ON was based on subacute onset of decreased visual acuity, a visual field defect, a relative afferent pupillary defect when unilateral, and gadolinium enhancement of the optic nerve on fat-suppressed magnetic resonance imaging (MRI) at onset.

Among the 49 patients, 35 eyes from 25 cases were enrolled in this study based on the following inclusion and exclusion criteria. The inclusion criteria were (1) patients who had received a Landolt ring-based, decimal visual acuity test, visual field test, and OCT measurements at least six months after the latest episode of ON, and (2) had the presence and titer of AQP4-Ab evaluated before treatments for new attacks of ON by an in-house cell-based assay in the Department of Neurology, Tohoku University, as described in elsewhere. [31] Exclusion criteria were (1) patients with other optic nerve disorders, glaucoma, diabetes, or multiple sclerosis, who met the revised diagnostic criteria by McDonald et al. [32], and (2) eyes with poor OCT image quality and unreliable visual field test results, as defined below.

We divided the subjects into two groups solely based on the presence or absence of AQP4-Ab. Demographic and clinical variables were compared between the AQP4-Ab-positive and -negative groups.

### Tested parameters and optical coherence tomography measurement

Best-corrected decimal visual acuity (VA) was converted to logMAR (minimal angle resolution) VA. Mean deviation (MD) of the Humphrey SITA-standard 30–2 (Carl Zeiss Meditec, Dublin, CA) test with false-positive, false-negative, and fixation loss scores < 25% was obtained. RTVue SD-OCT (software version 4.0.5.39; Optovue Inc., Fremont, CA) was used to measure cpRNFL and GCC thicknesses as previously reported.[24,33] In brief, the optic nerve head map protocol generated an RNFL thickness map along a circle 3.45 mm in diameter centered on the optic disc. A 3D disc protocol was used to register the edge of the optic nerve head. Only high-quality images were included, defined by a signal strength index > 30. The GCC protocol measured parameters within a circle of 6-mm diameter. The center of the GCC scan was shifted approximately 1-mm temporal to the fovea to improve the sampling of temporal peripheral nerve fibers. The OCT parameters measured in this study were the whole-retina average and quadrant (i.e., temporal, nasal, superior, and inferior) thicknesses of the cpRNFL, and the average, superior, and inferior thicknesses of the GCC.

The OCT measurements, visual field test, and acuity test were conducted on the same day for each patient.

### Statistical analysis

To compare patient demographics between the two groups, an unpaired t-test (parametric) or a Mann-Whitney U-test (non-parametric) were used for continuous variables depending on data distribution, while Fisher’s exact test was used for categorical variables.

Linear mixed effect models were used to investigate eye-based differences in MD, LogMAR VA, and OCT parameters between the two groups by setting the presence or absence of AQP4-Ab as the independent variable while adjusting for within-patient inter-eye correlation and sex. Given the significantly higher number of ON episodes in the AQP4 Ab-positive group, differences in visual function and OCT parameters were also tested in eyes with a single episode of ON.

Receiver operating characteristic curve (ROC) analysis was conducted to identify which parameter best discriminates AQP4 Ab-positive ON from AQP4 Ab-negative ON.

Scatter plots with Spearman rank correlation coefficients were used to visualize the associations between OCT parameters and both MD and LogMAR VA, between age and OCT parameters, and between number of ON episodes and the AQP4 Ab titer.

Generalized estimating equation (GEE) models with an ‘exchangeable’ working correlation matrix were used to investigate associations of OCT parameters (= the dependent variables) with predictive variables separately in the AQP4 Ab-negative and -positive groups while adjusting for within-patient, inter-eye, and intersex dependence. The predictive variables tested in both groups were age at OCT measurement, duration from the onset of the latest ON episode and OCT measurement, and ON episode number. In addition, an AQP4 Ab titer was also included as a predictive variable in the AQP4 Ab-positive group. Likewise, the associations of MD and LogMAR VA (= the dependent variables) with predictive variables were investigated in both groups using the GEE models. Although the predictive variables were essentially the same as above, OCT parameters were also included.

All statistical analyses were performed using SPSS (version 20.0, Japan IBM, Tokyo, Japan) with type I error for significance set at P<0.05. All tests should be understood and interpreted as constituting exploratory data analysis, so no previous power calculation or adjustments for multiple testing were made.

## Results

### Patient and eye characteristics

Of 35 eyes from 25 cases included in this study, 15 eyes from 13 cases were AQP4 Ab negative and 20 eyes from 12 cases were AQP Ab positive. Patient demographics are summarized in Table 1. There were no significant differences in age distribution, sex ratio, history of LETM, and performance of plasma exchange between the two groups. In contrast, the AQP4 Ab-positive group had a longer duration since the last ON episode, the number of steroid pulse therapy, and a higher proportion with a history of bilateral ON compared to the AQP4 Ab-negative group, in agreement with previous studies.[8,9]

**Table 1:** Patient-based characteristics.

Variables	AQP4 Ab negative groups (n = 13)		AQP4 Ab positive group (n = 12)		P-value*
	Range	Median (IQR)	Range	Median (IQR)	
**Age (Yrs)**	23~77	47.0 (34.8, 62.3)	10~64	43 (33.5, 48.5)	0.24
**Duration (Yrs)**	0.5~4.5	1.1 (0.58, 1.75)	0.5~10.8	3.4 (1.1, 8.4)	0.04
**AQP4 Ab titer**	N.A.	N.A.	16~65536	192 (75, 8448)	N.A.
**No. Steroid Pulse**	1~3	1(1, 3)	2~12	3(2.5, 6)	0.006
	**Ratio**		**Ratio**		
**Sex (Male/Female)**	3/10		2/10		1.00
**LETM (-/+)**	11/2		6/6		0.10
**Laterality (Uni-/Bi-lateral)**	11/2		4/8		0.02
**Plasma Exchange (-/+)**	12/1		8/4		0.16

AQP4 Ab, aquaporin 4 autoantibody; IQR, interquartile range; N.A., not applicable; LETM, longitudinally extensive transverse myelitis.

* Unpaired t-test was used to compare median age, Mann-Whitney U test for median duration, and Fisher's exact test for sex, laterality, and LETM ratios.

Eye-based characteristics of the two groups are summarized in Table 2. There was no significant difference in the number of ON episodes between the AQP4 Ab-positive and -negative groups, as previously reported.[8,9] Linear mixed effect models revealed worse MD and logMAR VA in the APQ4 Ab-positive group than the -negative group after adjusting for within-patient inter-eye correlation and sex, whereas there were no significant differences in average RNFL thickness, individual RNFL quadrant thicknesses, average GCC thickness, and hemifield GCC thicknesses between the two groups.

**Table 2:** Eye-based characteristics.

Variables	AQP4 Ab-negative groups (n = 15)		AQP4 Ab-positive group (n = 20)		P-value*
	Range	Median (IQR)	Range	Median (IQR)	
**MD (dB)**	-10.54~0.66	-2.48 (-4.36, -0.11)	-40.00~-1.69	-17.84 (-29.49, -4.79)	0.001
**logMAR VA**	-0.18~0.16	0.00 (-0.08, 0.00)	-0.18~3.00	0.05 (0.00, 1.15)	0.0001
**Average RNFL (μm)**	58.47~117.85	71.59 (64.87, 83.57)	50.55~97.19	66.03 (59.58, 87.21)	0.4
**Superior RNFL (μm)**	72.50~156.75	90.50 (82.25, 107.31)	67.42~132.50	82.13 (74.88, 102.13)	1
**Inferior RNFL (μm)**	76.25~144.00	94.75 (82.75, 113.00)	57.50~139.75	90.00 (84.75, 114.23)	0.13
**Temporal RNFL (μm)**	36.50~80.50	51.25 (39.88, 58.38)	29.25~60.25	45.00 (38.50, 52.13)	0.16
**Nasal RNFL (μm)**	42.75~90.25	58.00 (50.88, 63.19)	35.00~73.50	47.38 (44.07, 60.81)	0.27
**Average GCC (μm)**	59.53~98.59	68.41 (64.91, 79.33)	52.39~83.17	65.46 (59.21, 74.26)	0.15
**Superior GCC (μm)**	55.04~89.38	68.21 (65.13, 77.48)	53.85~82.72	68.10 (60.90, 74.85)	0.12
**Inferior GCC (μm)**	54.66~86.94	69.07 (61.26, 76.99)	50.71~83.63	65.13 (60.45, 75.85)	0.17
**ON Episodes**	1∼2	1 (1.0, 1.0)	1~5	1 (1.0, 2.5)	0.08

AQP4 Ab, aquaporin 4 autoantibody; IQR, interquartile range; ON, optic neuritis; MS, mean deviation; MAR, minimum resolution angle; VA, visual acuity; RNFL, retinal nerve fiber layer; GCC, ganglion cell complex.

* Mann-Whitney U-test was used to compare ON episode number, whereas a linear mixed effect model was used to compare all other variables with adjustment for within inter-eye and sex dependence.

**Fig 1:**
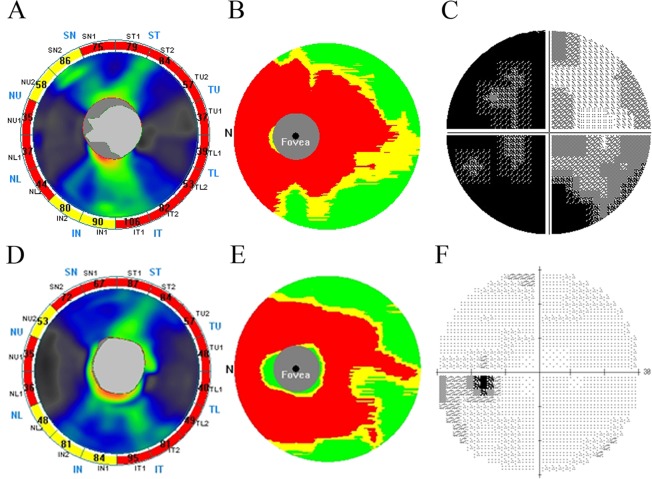
**Representative cases of aquaporin-4 antibody (AQP4 Ab)-positive (A−C) and -negative (D−F) optic neuritis in the left eye.** Shown are optic nerve head maps (A, D) and GCC significance maps (B, E) from RTVue optical coherence tomography analyses, and gray scales (C, F) of Humphrey visual field 30–2 tests. Tests were conducted 6.5 years and 4.5 years after the latest onset of optic neuritis in the seropositive and negative case, respectively.

Fig 1 shows representative optic nerve head maps (Fig 1A and 1D) and GCC significance maps (Fig 1B and 1D) of RTVue OCT and visual field gray scales (Fig 1C and 1F) for the left eye in an AQP4 Ab-positive case (Fig 1A–1C) and AQP4 Ab-negative case (Fig 1D–1F). The OCT and visual field were measured 6.5 and 4.5 years, respectively, after the onset of ON. Significantly reduced cpRNFL (Fig 1A and 1D) and GCC (Fig 1B and 1E) thicknesses from the database norms (in the RTVue software) are expressed in yellow (P < 0.05) and red (P < 0.01), respectively, whereas measures comparable with the database norms are expressed in green. Note that the areas of significantly reduced cpRNFL and GCC thickness were similar between the two eyes. The average cpRNFL thickness was 65.02 and 63.61 μm in the AQP4 Ab-positive and AQP4 Ab-negative cases, respectively, whereas the average GCC thickness was 64.32 and 66.67 μm in the positive and negative cases, respectively. In contrast, there was substantial disparity in the gray scale of the visual field between cases, with MDs of −21.08 and −2.73 dB in the seropositive and seronegative cases, respectively. These two representative cases clearly illustrate that despite similar reductions in cpRNFL and GCC thickness, the visual field defect was more severe in the anti-AQP4 Ab-positive case. The visual field defect was mild in the anti-AQP4 Ab-negative case.

Raw OCT data from all of the 25 cases (cases 1 through 25) are listed in S1 Fig.

In line with these findings, ROC analyses also demonstrated that MD and logMAR VA could discriminate AQP4 Ab-positive ON from AQP4 Ab-negative ON, whereas only the OCT parameter nasal quadrant RNFL thickness could discriminate between these two groups (Table 3).

**Table 3:** Receiver Operating Characteristic Curve Analyses for Factors Discriminating AQP4 Ab Positivity.

Variables	AUC	SE	95% CI	P-value
**MD**	0.88	0.06	0.73 ~ 0.97	<0.0001
**logMAR VA**	0.76	0.08	0.58 ~ 0.89	0.001
**Average RNFL**	0.59	0.10	0.41 ~ 0.75	0.37
**Superior RNFL**	0.61	0.10	0.43 ~ 0.77	0.26
**Inferior RNFL**	0.53	0.10	0.35 ~ 0.70	0.80
**Temporal RNFL**	0.64	0.10	0.46 ~ 0.80	0.13
**Nasal RNFL**	0.69	0.09	0.51 ~ 0.83	0.04
**Average GCC**	0.64	0.10	0.46 ~ 0.79	0.15
**Superior GCC**	0.58	0.10	0.41 ~ 0.75	0.40
**Inferior GCC**	0.58	0.10	0.40 ~ 0.74	0.44

AUC, area under the ROC curve; SE, standard error; CI, confidence interval; MD, mean deviation; MAR, minimum resolution angle; VA, visual acuity; RNFL, retinal nerve fiber layer; GCC, ganglion cell complex.

**Fig 2:**
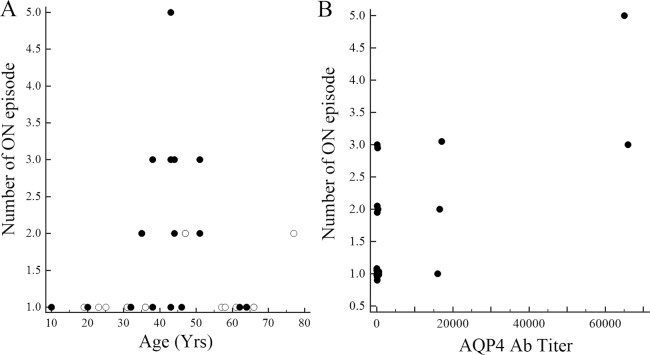
**Scatter plots of number of optic neuritis (ON) episodes against age (A) and titer of the anti-aquaporin 4 autoantibody (AQP4 Ab) (B).** Open circles indicate eyes with AQP4 Ab-negative ON, whereas closed circles indicate those with antibody-positive ON. Spearman rank correlation coefficient (95% confidence interval, P-value) between number of ON episodes and AQP4 Ab titer was 0.46 (0.02 to 0.75, 0.04).

Fig 2 depicts scatterplots of the ON episode number against age (Fig 2A) for the eyes with (closed circles) and without (open circles) AQP4 Ab and against AQP4 Ab titer for the eyes with the antibody (Fig 2B). Repeated episodes of ON were more common in middle-age patients (Fig 2A), and there was a weak but significant positive correlation between AQP4 Ab titer and the number of ON episodes (Fig 2B).

Table 4 summarizes the Spearman rank correlation coefficients of AQP4 Ab titer against clinical parameters for the eyes with AQP4 Ab. There were significant inverse correlations between AQP4 Ab titer and average RNFL, temporal RNFL, average GCC, superior GCC, and inferior GCC thickness, while there was no significant correlation between titer and MD or logMAR VA. Given the significant positive correlation between the number of ON episodes and AQP4 Ab titer (Fig 2B), the correlations of OCT parameters with AQP4 Ab titer are thought to depend on the number of ON recurrences.

**Table 4:** Retinal Structure Correlations with Ab Titer in the AQP4 Ab-Positive Group.

Variables	r_s_	95% CI	P-value
**MD**	-0.31	-0.66 to 0.15	0.18
**logMAR VA**	0.32	-0.14 to 0.67	0.16
**Average RNFL**	-0.24	-0.62 to 0.23	0.31
**Temporal RNFL**	-0.55	-0.80 to -0.14	0.01
**Superior RNFL**	-0.18	-0.58 to 0.29	0.45
**Nasal RNFL**	-0.13	-0.54 to 0.33	0.59
**Inferior RNFL**	-0.17	-0.57 to 0.30	0.48
**Average GCC**	-0.47	-0.75 to -0.03	0.04
**Superior GCC**	-0.46	-0.75 to -0.02	0.04
**Inferior GCC**	-0.55	-0.80 to -0.14	0.01
**ON episode**	0.46	0.02 to 0.75	0.04

r_s_, Spearman rank correlation; CI, confidence interval; AQP4 Ab, aquaporin 4 autoantibody; MD, mean deviation of Humphrey Visual Field; MAR, minimum resolution angle; VA, best-corrected visual acuity; RNFL, retinal nerve fiber layer thickness; GCC, ganglion cell complex; ON, optic neuritis.

### Factors associated with OCT parameters

**Fig 3:**
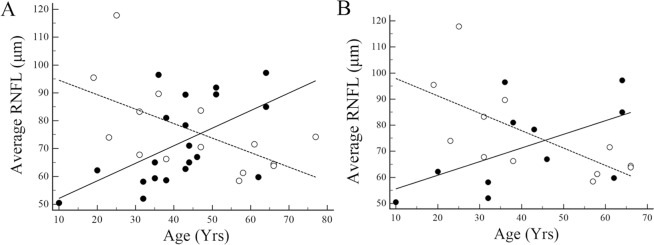
**Associations of circumpapillary retinal nerve fiber layer (RNFL) average thickness with age in all eyes (A) and eyes with a single episode of optic neuritis (B).** Open circles and dotted lines indicate eyes with aquaporin-4 antibody (AQP4 Ab)-negative optic neuritis, whereas closed circles and solid lines indicate eyes with antibody-positive optic neuritis. Spearman rank correlation coefficients (95% confidence interval, P-value) for the AQP4 Ab-negative and -positive groups were -0.57 (-0.84 to -0.08, 0.03) and 0.63 (0.27 to 0.84, 0.003), respectively, in the all eyes (A), and -0.74 (-0.92 to -0.28, 0.006) and 0.67 (0.12 to 0.91, 0.024), respectively, in eyes with a single episode of optic neuritis (B).

Fig 3 presents scatter plots of average cpRNFL thickness against age at OCT measurements for all eyes (Fig 3A) and eyes with a single ON episode (Fig 3B). There was a significantly negative correlation between average cpRNFL thickness and age in the AQP4 Ab-negative group and a significant positive correlation between average cpRNFL thickness and age in the positive group, both for all eyes and eyes with a single ON episode.

To evaluate the impact of AQP4 Ab presence, age, duration from the latest ON episode, and clinical examination parameters (including OCT, visual field, acuity test results, and number of ON episodes) on OCT parameters, GEE regression analyses were conducted with adjustment for within-patient inter-eye and sex dependence. Tables 5 and 6 list the coefficient estimates for GEE (B), standard error of B, and probability of significance in patients without, and with, AQP4 Ab, respectively.

**Table 5:** Generalized Estimating Equation Regression Analyses for Optical Coherence Tomography Parameters in Aquaporin-4 Antibody-Negative Eye.

Dependent Variables	Predictor Variables							
	Age		Duration		ON Episodes		Interaction	
	B (SE)	P	B (SE)	P	B (SE)	P	B (SE)	P
**Average RNFL**	-0.69 (0.27)	0.01	-14.35 (0.27)	0.01	-6.91 (5.93)	0.24	0.21 (0.08)	0.005
**Temporal RNFL**	-0.49 (0.23)	0.035	-9.61 (0.23)	0.035	0.29 (4.11)	0.94	0.16 (0.07)	0.013
**Nasal RNFL**	-0.53 (0.21)	0.013	-11.79 (0.21)	0.013	-5.70 (5.27)	0.28	0.19 (0.06)	0.003
**Superior RNFL**	-1.05 (0.42)	0.012	-19.89 (0.42)	0.012	-2.87 (9.55)	0.76	0.32 (0.12)	0.008
**Inferior RNFL**	-0.69 (0.31)	0.025	-16.36 (0.31)	0.025	-18.74 (6.94)	0.007	0.23 (0.08)	0.006
**Average GCC**	-0.67 (0.16)	0.00	-11.44 (0.16)	0.00	-1.12 (3.30)	0.74	0.19 (0.04)	0.00
**Superior GCC**	0.49 (0.24)	0.038	7.89 (0.24)	0.038	-0.14 (1.87)	0.94	-0.12 (0.08)	0.13
**Inferior GCC**	0.22 (0.23)	0.33	5.10 (0.23)	0.33	-4.41 (2.47)	0.07	-0.05 (0.07)	0.55

SE, standard error of B; ON, optic neuritis; RNFL, retinal nerve fiber layer thickness; GCC, ganglion cell complex.

**Table 6:** Generalized Estimating Equation Regression Analyses for Optical Coherence Tomography Parameters in Aquaporin-4 Antibody-Positive Eyes.

Dependent Variables	Predictor Variables									
	AQP4 Ab titer		Age		Duration		ON Episodes		Interaction	
	B (SE)	P	B (SE)	P	B (SE)	P	B (SE)	P	B (SE)	P
**Average RNFL**	0.001 (0.0001)	0.00	0.68 (0.24)	0.006	1.08 (0.72)	0.13	-7.00 (2.87)	0.02	-1.87×10^−7^ (1.06×10^−7^)	0.08
**Temporal RNFL**	-9.82×10^−5^ (9.71×10^−5^)	0.31	0.22 (0.16)	0.16	-0.23 (0.16)	0.68	-2.60 (1.96)	0.18	9.52×10^−8^ (6.04×10^−8^)	0.12
**Nasal RNFL**	0.00 (0.0001)	0.17	0.66 (0.08)	0.00	0.30 (0.23)	0.20	9.53 (1.61)	0.00	-5.81×10^−8^ (4.95×10^−8^)	0.24
**Superior RNFL**	0.001 (0.0002)	0.00	1.07 (0.19)	0.00	1.64 (1.01)	0.10	-13.60 (3.00)	0.00	-4.92×10^−7^ (9.53×10^−8^)	0.00
**Inferior RNFL**	0.00 (0.0002)	0.035	0.37 (0.47)	0.43	1.06 (1.56)	0.46	-9.18 (8.06)	0.26	-1.16×10^−7^ (2.83×10^−7^)	0.68
**Average GCC**	-0.001 (-2.00×10^−5^)	0.00	0.51 (0.05)	0.00	-0.61 (0.03)	0.00	11.02 (1.15)	0.00	4.71×10^−7^ (9.34×10^−9^)	0.00
**Superior GCC**	-0.001 (3.10×10^−5^)	0.00	0.46 (0.02)	0.00	-0.62 (0.05)	0.00	9.68 (0.30)	0.00	5.82×10^−7^ (1.17×10^−8^)	0.00
**Inferior GCC**	-0.001 (6.13×10^−6^)	0.00	0.57 (0.02)	0.00	-0.56 (0.01)	0.00	9.88 (0.34)	0.00	3.78×10^−7^ (3.01×10^−9^)	0.00

AQP4 Ab, aquaporin-4 antibody; ON, optic neuritis; Interaction, interactions of listed predictor variables with visual functions; SE, standard error of B; RNFL, retinal nerve fiber layer thickness; GCC, ganglion cell complex.

In patients without AQP4 Ab, age and duration were significantly negatively associated with all OCT parameters tested except superior and inferior GCC thickness, while the number of ON episodes was not significantly associated with any OCT parameter except inferior RNFL and inferior GCC thicknesses (Table 5). These predictive variables showed significant interactions with all other OCT parameters except superior and inferior GCC thickness. Therefore, the older the patient and the longer the duration of disease, the thinner the OCT parameters in ON patients without AQP4 Ab. The relatively weak association of OCT parameters with the number of ON episodes was probably due to the relatively rare incidence of recurrence in this group.

In AQP4 Ab-positive patients, there were significant positive associations between age and all OCT parameters except temporal and inferior quadrant RNFL thickness (Table 6). Although the duration and number of ON episodes were also significantly associated with some OCT parameters, the direction of this association depended on the specific combinations of parameters (Table 6). The AQP4 Ab titer also showed statistically significant associations with all OCT parameters except temporal and nasal RNFL thickness. However, the magnitudes of correlations were weak (Table 6). The superior RNFL and average and hemifield GCC thickness had significant interactions with other tested predictive parameters. Therefore, these variables showed complex interactive effects on the development of structural changes in the retina.

These results are essentially in accordance with the scatter plots and Spearman rank correlation analyses shown in Fig 3 and Table 4.

### Factors associated with visual functions

**Fig 4:**
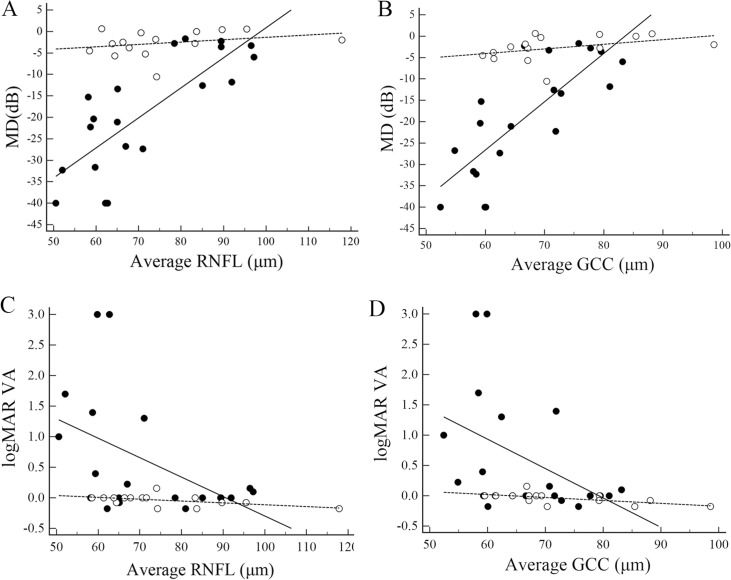
Structure−function relationships in all eyes. Scatter plots of mean deviation (MD) against circumpapillary retinal nerve fiber layer (RNFL) average thickness (A), MD against ganglion cell complex (GCC) average thickness (B), logMAR visual acuity (VA) against circumpapillary RNFL average thickness (C), and VA against GCC average thickness (D). Open circles and dotted lines indicate eyes with aquaporin 4 antibody (AQP4 Ab)-negative optic neuritis, whereas closed circles and solid lines indicate eyes with antibody-positive optic neuritis. Spearman rank correlation coefficients (95% confidence interval, P-value) for the AQP4 Ab-negative and positive-groups were 0.33 (-0.22 to 0.72, 0.23) and 0.74 (0.44 to 0.89, 0.0002), respectively, between MD and average RNFL thickness (A), 0.49 (-0.03 to 0.80, 0.06) and 0.73 (0.43 to 0.89, 0.0002), respectively, between MD and average GCC thickness (B), -0.57 (-0.84 to -0.07, 0.03) and -0.40 (-0.71 to 0.06, 0.08), respectively, between logMAR VA and average RNFL thickness (C), and -0.69 (-0.89 to -0.27, 0.005) and -0.52 (-0.78 to -0.10, 0.02), respectively, between logMAR VA and average GCC thickness (D).

**Fig 5:**
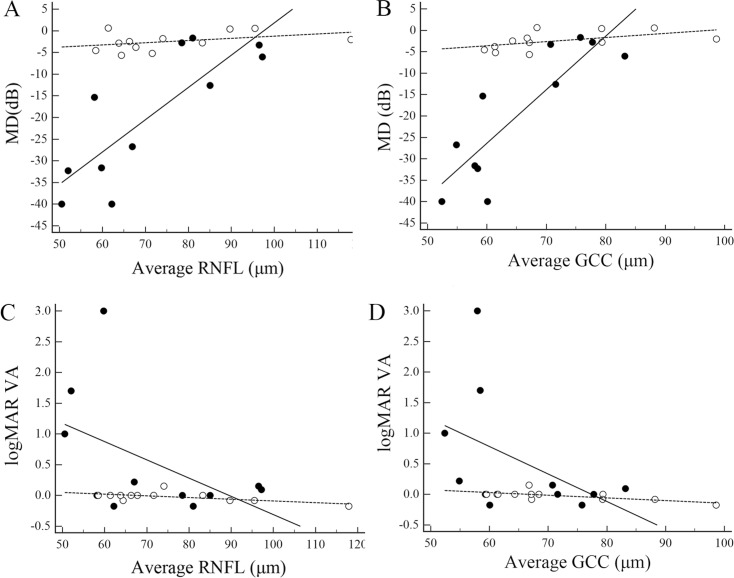
Structure−function relationships in eyes with single-episode optic neuritis. Scatter plots of mean deviation (MD) against circumpapillary retinal nerve fiber layer (RNFL) average thickness (A), MD against ganglion cell complex (GCC) thickness (B), logMAR visual acuity (VA) against circumpapillary RNFL average thickness (C), and VA against GCC average thickness (D). Open circles and dotted lines indicate eyes with aquaporin 4 antibody (AQP4 Ab)-negative optic neuritis, whereas closed circles and solid lines indicate eyes with antibody-positive optic neuritis. Spearman rank correlation coefficients (95% confidence interval, P-value) for the antibody-negative and -positive groups were 0.39 (-0.24 to 0.79, 0.22) and 0.75 (0.27 to 0.93, 0.008), respectively, between MD and average RNFL thickness (A), 0.58 (0.01 to 0.87, 0.05) and 0.77 (0.32 to 0.94, 0.006), respectively, between MD and average GCC thickness (B), -0.47 (-0.82 to 0.14, 0.12) and -0.39 (-0.80 to 0.28, 0.24), respectively, between logMAR VA and average RNFL thickness (C), and -0.65 (-0.89 to -0.11, 0.02) and -0.62 (-0.89 to -0.02, 0.04), respectively, between logMAR VA and average GCC thickness (D).

Figs 4 and 5 depict scatter plots of visual functions against average cpRNFL and GCC thickness for all eyes and eyes with a single ON episode, respectively. There were significant positive correlations between MD and both cpRNFL and GCC thickness in the AQP4 Ab-positive ON eyes irrespective of whether there were multiple or a single episode of ON. In contrast, neither the whole population of AQP4 Ab-negative ON eyes nor those with a single ON episode showed a significant correlation between MD and cpRNFL thickness or MD and GCC thickness. Alternatively, logMAR VA was significantly correlated with average RNFL thickness only in the AQP4 Ab-negative eyes with a single ON episode. In both groups, logMAR VA was significantly correlated with the average GCC thickness independent of the number of ON episodes.

To exclude within-patient inter-eye dependence and account for interactions among variables, GEE regression analyses were conducted for factors associated with MD or logMAR VA in AQP4 Ab-negative and -positive eyes. In AQP4 Ab-negative eyes, there was no association between MD and any OCT parameter or number of ON episodes, whereas age and duration showed significant inverse correlations with MD. Thus, older age and longer disease duration were associated with worse MD in this group of eyes (Table 7). In contrast, all OCT parameters except inferior quadrant RNFL and average GCC thicknesses were negatively (albeit weakly) correlated with logMAR VA. The number of ON episodes was associated with logMAR VA only when average, nasal, and inferior RNFL thicknesses as well as average GCC thickness were included as predictive variables. Other variables had no significant correlations with logMAR VA. Therefore, inner retinal structural changes were not correlated with the visual field defect and showed only limited impact on visual acuity in AQP4Ab-negative eyes.

**Table 7:** Generalized Estimating Equation Regression Analyses for Factors associated with Visual Function in Aquaporin-4 Antibody-Negative Eyes.

Dependent Variables	Predictor Variables										
	OCT Parameters			Age		Duration		ON Episodes		Interaction	
		B (SE)	P	B (SE)	P	B (SE)	P	B (SE)	P	B (SE)	P
**MD**	Average RNFL	-0.05 (0.04)	0.22	-0.21 (0.05)	0.00	-2.53 (0.67)	0.00	-1.65 (2.07)	0.43	0.001 (0.0002)	0.00
	Temporal RNFL	-0.59 (0.05)	0.25	-0.19 (0.05)	0.00	-2.21 (0.58)	0.00	-1.37 (1.96)	0.48	0.001 (0.0002)	0.00
	Nasal RNFL	-0.13 (0.05)	0.01	-0.23 (0.05)	0.00	-2.92 (0.64)	0.00	-1.69 (1.81)	0.35	0.001 (0.0002)	0.00
	Superior RNFL	-0.05 (0.02)	0.08	-0.21 (0.05)	0.00	-2.47 (0.61)	0.00	-1.54 (2.20)	0.45	0.001 (0.0001)	0.00
	Inferior RNFL	-0.007 (0.04)	0.87	-0.20 (0.05)	0.00	-2.29 (0.63)	0.00	-1.22 (2.06)	0.55	0.001 (0.0001)	0.00
	Average GCC	-0.07 (0.07)	0.31	-0.22 (006)	0.001	-2.62 (0.80)	0.001	-1.47 (1.97)	0.45	0.001 (0.0002)	0.00
	Superior GCC	-0.02 (0.03)	0.48	-0.20 (0.05)	0.00	-2.49 (0.68)	0.00	-1.58 (2.14)	0.46	0.001 (0.0002)	0.00
	Inferior GCC	0.20 (0.04)	0.58	-0.21 (0.05)	0.00	-2.80 (0.70)	0.00	-1.27 (2.03)	0.53	0.001 (0.0002)	0.00
**logMAR VA**	Average RNFL	-0.03 (0.001)	0.003	0.00 (0.001)	0.86	-0.06 (0.02)	0.73	-0.10 (0.05)	0.03	2.78×10^−7^ (4.44×10^−6^)	0.95
	Temporal RNFL	-0.003 (0.001)	0.02	0.01 (0.001)	0.62	0.003 (0.01)	0.81	-0.84 (0.05)	0.09	2.93×10^−7^ (5.22×10^−6^)	0.96
	Nasal RNFL	-0.06 (0.002)	0.00	0.00 (0.001)	0.72	-0.23 (0.02)	0.25	-0.11 (0.03)	0.00	5.18×10^−6^ (6.62×10^−6^)	0.43
	Superior RNFL	-0.002 (0.0008)	0.002	1.33×10^−5^ (0.001)	0.99	-0.006 (0.02)	0.76	-0.88 (0.04)	0.50	2.70×10^−7^ (3.68×10^−6^)	0.94
	Inferior RNFL	-0.002 (0.001)	0.10	0.01 (0.001)	0.60	0.00 (0.02)	0.99	-0.12 (0.06)	0.04	-3.03×10^−7^ (3.51×10^−6^)	0.93
	Average GCC	-0.07 (0.003)	0.009	-0.002 (0.002)	0.38	-0.03 (0.03)	0.38	-0.09 (0.42)	0.04	-7.93×10^−6^ (7.84×10^−6^)	0.31
	Superior GCC	0.04 (0.003)	0.16	0.002 (0.002)	0.43	0.03 (0.03)	0.36	-0.67 (0.48)	0.17	-8.44×10^−6^ (9.22×10^−6^)	0.36
	Inferior GCC	0.003 (0.002)	0.16	0.002 (0.002)	0.28	0.03 (0.03)	0.32	-0.06 (0.05)	0.18	-8.29×10^−6^ (7.55×10^−6^)	0.27

OCT, optical coherence tomography; ON, optic neuritis; Interaction, interactions of listed predictor variables with visual functions; SE, standard error of B; MD, mead deviation; RNFL, retinal nerve fiber layer; GCC, ganglion cell complex; VA, visual acuity

Table 8 summarizes GEE analyses of associations between visual functions and predictive variables in AQP4Ab-positive eyes. In this group, all OCT parameters except superior GCC thickness were significantly correlated with MD depending on AQP4 Ab titer, age, duration, and number of ON episodes. These associations were also observed for logMAR VA, although the strengths of associations were weaker than those with MD. These findings also support the structure−function relationships in AQP4 Ab-positive patients revealed by the scatter plots and Spearman rank correlation analyses of Figs 4 and 5 and Table 4.

**Table 8:** Generalized Estimating Equation Regression Analyses for Factors associated with Visual Function in Aquaporin-4 Antibody-Positive Eyes.

Dependent Variables	Predictor Variables												
	OCT Parameters			AQP4 Ab Titer		Age		Duration		ON Episodes		Interaction	
		B (SE)	P	B (SE)	P	B (SE)	P	B (SE)	P	B (SE)	P	B (SE)	P
**MD**	Average RNFL	0.81 (0.11)	0.00	0.001 (0.00)	0.02	0.14 (0.18)	0.44	0.83 (0.73)	0.26	-1.26 (3.01)	0.68	-8.03×10^−9^ (2.98×10^−8^)	0.007
	Temporal RNFL	2.08 (0.40)	0.00	0.001 (0.00)	0.00	0.12 (0.26)	0.64	2.05 (0.86)	0.02	-1.65 (1.64)	0.32	-1.34×10^−8^ (1.27×10^−9^)	0.00
	Nasal RNFL	-0.21 (0.07)	0.002	-0.001 (1.82×10^−5^)	0.00	0.52 (0.05)	0.00	-0.33 (0.04)	0.00	8.66 (0.51)	0.00	1.11×10^−8^ (1.13×10^−10^)	0.00
	Superior RNFL	-0.42 (0.02)	0.00	-0.002 (3.30×10^−5^)	0.00	1.07 (0.04)	0.00	-0.40 (0.00)	0.00	9.65 (0.14)	0.00	9.16×10^−9^ (1.88×10^−10^)	0.00
	Inferior RNFL	-0.11 (0.27)	0.00	-0.002 (5.78×10^−5^)	0.00	0.56 (0.04)	0.00	-0.45 (0.02)	0.00	8.44 (0.31)	0.00	7.15×10^−9^ (2.68×10^−10^)	0.00
	Average GCC	-0.39 (0.03)	0.00	-0.002 (4.17×10^−5^)	0.00	0.58 (0.02)	0.00	-0.63 (0.03)	0.00	10.91 (0.41)	0.00	1.29×10^−8^ (2.33×10^−10^)	0.00
	Superior GCC	0.10 (0.49)	0.85	-0.001 (0.0007)	0.07	0.34 (0.24)	0.15	-0.33 (0.31)	0.29	5.71 (5.11)	0.26	8.41×10^−9^ (4.32×10^−9^)	0.05
	Inferior GCC	-1.17 (0.14)	0.00	-0.003 (0.0002)	0.00	1.04 (0.08)	0.00	-1.05 (0.08)	0.00	18.13 (1.42)	0.00	1.86×10^−8^ (9.16×10^−10^)	0.00
**logMAR VA**	Average RNFL	-0.01 (0.01)	0.28	1.96×10^−5^ (1.23×10^−5^)	0.11	-0.01 (0.01)	0.29	-0.05 (0.01)	0.00	0.04 (0.09)	0.64	5.88×10^−12^ (7.68×10^−11^)	0.94
	Temporal RNFL	-0.07 (0.01)	0.00	-1.68×10^−5^ (6.74×10^−5^)	0.01	0.03 (0.01)	0.00	-0.09 (0.01)	0.00	0.38 (0.06)	0.00	3.37×10^−10^ (6.18×10^−10^)	0.00
	Nasal RNFL	0.05 (0.008)	0.00	4.12×10^−5^ (2.12×10^−6^)	0.00	-0.06 (0.01)	0.00	-0.06 (0.00)	0.00	-0.48 (0.06)	0.00	-5.24×10^−11^ (1.32×10^−11^)	0.00
	Superior RNFL	0.07 (0.006)	0.00	0.00 (9.36×10^−6^)	0.00	-0.15 (0.01)	0.00	-0.04 (0.00)	0.00	-0.54 (0.04)	0.00	-6.56×10^−10^) (5.23×10^−11^)	0.00
	Inferior RNFL	0.02 (0.002)	0.00	7.83×10^−5^ (5.18×10^−6^)	0.00	-0.06 (0.00)	0.00	-0.03 (0.00)	0.00	-0.39 (0.03)	0.00	-2.54×10^−10^ (2.40×10^−11^)	0.00
	Average GCC	0.06 (0.01)	0.00	0.00 (1.40×10^−5^)	0.00	-0.06 (0.01)	0.00	-0.007 (0.01)	0.50	-0.68 (0.14)	0.00	-5.45×10^−10^ (7.88×10^−11^)	0.00
	Superior GCC	0.06 (0.08)	0.45	0.00 (0.0001)	0.31	-0.05 (0.04)	0.18	-0.005 (0.05)	0.93	-0.63 (0.86)	0.47	-6.31×10^−10^ (7.26×10^−10^)	0.39
	Inferior GCC	0.18 (0.03)	0.00	0.00 (3.68×10^−5^)	0.00	-0.13 (0.02)	0.00	0.06 (0.02)	0.00	-1.83 (0.33)	0.00	-1.32×10^−9^ (2.06×10^−10^)	0.00

OCT, optical coherence tomography; AQP4 Ab, aquaporin-4 antibody; ON, optic neuritis; Interaction, interactions of listed predictor variables with visual functions; SE, standard error of B; MD, mead deviation; RNFL, retinal nerve fiber layer; GCC, ganglion cell complex; VA, visual acuity.

## Discussion

Only a few studies have evaluated the prognostic impact of AQP4 Ab in patients with ON.[7–10] According to Jarius et al.,[8] AQP4 Ab-positive acute monophasic ON led to more frequent complete bilateral or unilateral blindness compared to AQP4 Ab-negative cases. However, their AQP4 Ab titer did not correlate with disease severity. Later, the same group reported that there were no significant differences in age at onset, time to relapse, annualized relapse rates, and outcome from relapse between seropositive and negative cases.[9] However, these studies did not measure retinal structure by OCT and thus did not identify the impact of seropositivity on the retinal structure−function relationship.

This study demonstrated the impact of AQP4 Ab presence on inner retinal structure and functional outcomes in eyes with ON based on the following findings. First, although RNFL and GCC thicknesses were distributed roughly within a similar range, the eyes from patients with AQP4 Ab showed significantly poorer visual functional outcomes than those without the antibody. Second, when adjusted for within-patient inter-eye dependence, the changes in inner retinal structure were associated with AQP4 Ab presence, age, and number of ON episodes. There were also significant interactions between changes in inner retinal structure and these clinical parameters. In eyes without AQP4 Ab, most OCT parameters became thinner with age, whereas in eyes with AQP4 Ab, younger age was associated with thinning of most OCT parameters. Third, irrespective of the number of ON episodes, MD decline, and to a lesser extent VA decline, was proportional to RNFL and GCC thinning only in eyes with AQP4 Ab. In contrast, there was no clear structure−function relationship in eyes without AQP4 Ab. These findings suggest a critical role for AQP4 Ab or AQP4 Ab-positive serum in optic nerve degeneration.

The comparable changes in cpRNFL between AQP4 Ab positive and negative eyes among ON patients are in agreement with a previous study that used the older TD OCT to measurer RNFL thickness.[34] Although the number of ON recurrences had a significant negative impact on retinal structural changes as previously reported,[30] this did not directly reflect the magnitude of final visual impairment. OCT measures thickness of circumpapillary RNFL and inner macular retinal structures, which comprise not only RGCs but also retinal glial (Müller cells and astrocytes) and vascular components.[35] Thinning of these parameters reflects not only loss of RGCs but also retinal tissue remodeling, which has been implicated in glaucomatous optic neuropathy.[35] Given that visual function is ultimately dependent on RGC viability and activity, the disparity between OCT parameters and visual function in eyes without AQP4 Ab suggest that thinning of RNFL and GCC in this group is primarily due to retinal remodeling rather than RGC loss. In contrast, the structure−function relationship was clear in eyes with APQ4 Ab, so thinning of OCT parameters may directly reflect RGC loss rather than retinal tissue remodeling. Intriguingly, the AQP4 Ab titer in seropositive eyes was correlated more strongly with GCC than cpRNFL thickness (Tables 4 and 6). Given that the RGC population is dense in the macular region, corresponding to the area of GCC measurement, it is reasonable to speculate that these parameters are more likely to reflect AQP4 Ab-induced RGC loss. On the other hand, the existence of a few AQP4 Ab-positive cases with unilateral involvement suggest that both systemic and local environments contribute to ON expression.

Costello et al. previously reported a threshold RNFL thickness below which predicted persistent visual dysfunction in multiple sclerosis patients. [36,37] In this study, however, it was difficult to define a clear cpRNFL or GCC thickness cut-off to predict final visual functional outcome in AQP4 Ab-positive eyes because structure−function relationships were near linear rather than polynomial. Nonetheless, MD was substantially reduced if the average RNFL and GCC were thinner than 75 μm, similar to multiple sclerosis.[36]

Another intriguing finding of this study is the influence of age on structural changes in the retina. Given that the cpRNFL and inner macular retinal structures become thinner with age in normal eyes,[38, 39] the age-dependent decline of OCT parameters in eyes without AQP4 Ab may merely reflect physiological aging rather than disease-related pathological changes. Alternatively, eyes in younger patients with AQP4 Ab and ON exhibited significantly thinner RNFLs and GCCs than older AQP4 Ab-positive patients. This was not related to the number of ON episodes, because thickness showed a bell-shaped distribution with age. Further, the AQP4 Ab titer could not account for the more severe damage of the inner retinal structure in younger AQP4 Ab-positive cases. It is known that monophasic NMO is more likely to affect patients at younger ages and at lower AQP4Ab titers, and that additional factors such as compliments are involved in the development of ON in patients with AQP4 Ab. Kitley et al. reported that early-onset seropositive NMOSD patients were more likely to develop blindness.[10] Taken together, more destructive immune responses may be triggered in young patients exposed to AQP4 Ab even for the first time.

The correlations of VA with changes in inner retinal structure were weaker than the correlations between MD and retinal structure changes, probably because VA reflects the function of a very narrow area of fovea within the retina, whereas visual field reflects the function of the wider retina. Therefore, VA may be maintained if foveal function is preserved even when the remaining broader macular region is damaged. However, VA was poor if the average cpRNFL or GCC thickness was reduced below 60 μm in eyes with AQP4 Ab, which was in good agreement with the findings of Noval et al.[15] and Bennett et al.[21]

Limitations of this study include retrospective design and small sample size. Because this study was limited to a Japanese population, it is unclear whether these findings can be generalized to patients across other ethnicities. Eyes too severely damaged to undergo the Humphrey visual field test were excluded. Evidence is accumulating that shows an association of anti-myelin oligodendrocyte glycoprotein (MOG) antibody with the development of ON. Recent studies have demonstrated that approximately half of AQP4-Ab seronegative patients with ON had the MOG antibody.[40, 41] The presence of MOG antibody is thought to be a better prognostic factor for the outcome after ON,[40, 41] although the significance of the MOG antibody in the pathogenesis of the optic nerve degeneration is yet unclear. Due to the retrospective design, we could not evaluate the involvement of the MOG antibody in the groups of patients in the present study. In addition, the length of the optic nerve MRI lesion at the acute phase of ON was not evaluated. A recent study has shown that it may be better correlated with the final visual prognosis than OCT parameters measured at the chronic phase, [42] although that study examined visual acuity, but not visual field, which might have led to the weaker association of the OCT parameters than the MRI findings with the visual function. Microcystic macular edema (MME) has been reported to appear in the inner nuclear layer in a fraction of patients with NMO, [21] which was not assessed in the present study. However, the absence of MME evaluation likely does not affect our conclusions because the primary OCT parameters measured in this study, macular inner retinal layer (GCC) thickness and RNFL thickness, are not associated with the development of MME. Furthermore, the effects of treatment option and intensity were not evaluated. Although the number of patients who received PE was not statistically different between those with and without AQP4 Ab, the total number of steroid pulse therapy in those who had the AQP4 Ab was significantly more than that in those who did not have the antibody. However, the choice of treatment for each patient was determined by the response of visual function to treatment. If the first-line steroid pulse therapy failed to ameliorate or deteriorated visual function, PE was immediately conducted. In other words, if visual function rapidly improved in response to steroid therapy, intensive treatments, such as PE, was not included. In addition, steroid pulse therapy was repeated only in cases of optic neuritis relapse. Therefore, the use of steroid pulse therapy and whether or not PE was applied was highly dependent on the responsiveness to steroid therapy, severity of visual dysfunction, and number of relapses. Thus, the choice of treatment and these variables were interrelated. Conversely, the number of optic neuritis relapses and degree of visual dysfunction differed between the bilateral eyes in some patients with bilateral involvement. The intensity of treatment was determined on the basis of the eye status with a greater number of relapses or more severity. Therefore, for eyes with a lower frequency of relapse or less severity in the same patients, such an aggressive treatment may be beyond indication. Therefore, it is not appropriate to include the treatment option (the number of steroid pulse and presence or absence of PE) as an explanatory variable from both statistical and clinical viewpoints.

In conclusion, the presence of AQP4 Ab not only led to worse visual function outcomes, but also presumably had a significant impact on RGC loss, because RGCs are included in the inner retinal structure detected in OCT and transmit visual information. The non-neuronal elements, which are other constituents of the OCT-detected inner retinal structure, are likely to be predominantly reduced in the eyes of patients without AQP4 Ab, as indicated by good visual outcomes. We believe that such findings provide new insights into the understanding of the neuronal degeneration process of optic neuritis with and without AQP4 Ab.

## Supporting information

S1 Fig Optic Nerve Head Maps and GCC Significance Maps from RTVue Optical Coherence Tomography of All the 25 Cases. (PPTX)
