# Supplementary material for: Impact of the anti-aquaporin-4 autoantibody on inner retinal structure, function and structure-function associations in Japanese patients with optic neuritis
Source: PLoS One. 2017 Feb 15;12(2):e0171880. doi: 10.1371/journal.pone.0171880 (PMC5310877; doi:10.1371/journal.pone.0171880)
Supplement: S1 Fig — (PPTX) [file pone.0171880.s001.pptx]

## Slide 1
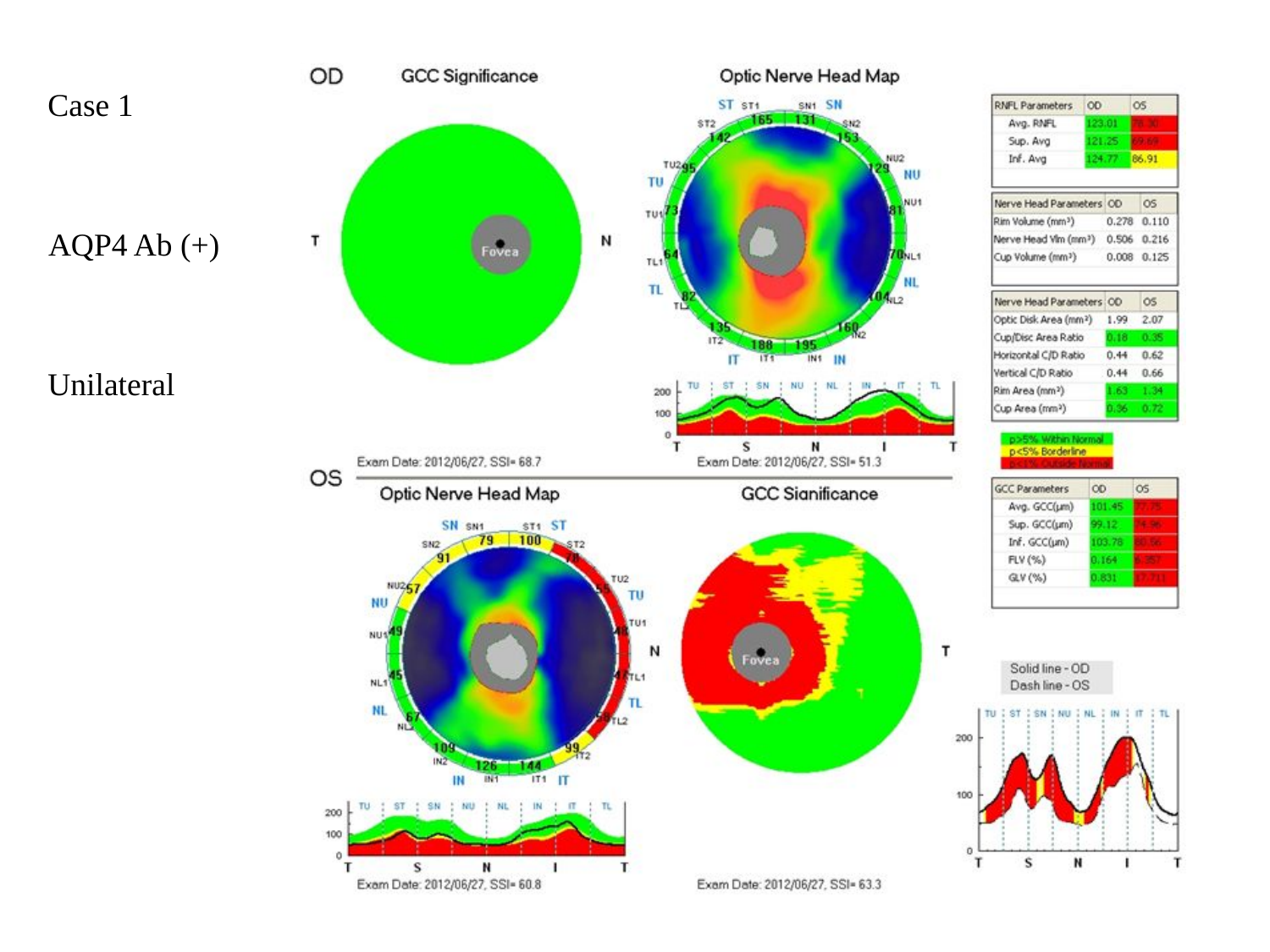

Case 1
AQP4 Ab (+)
Unilateral

## Slide 2
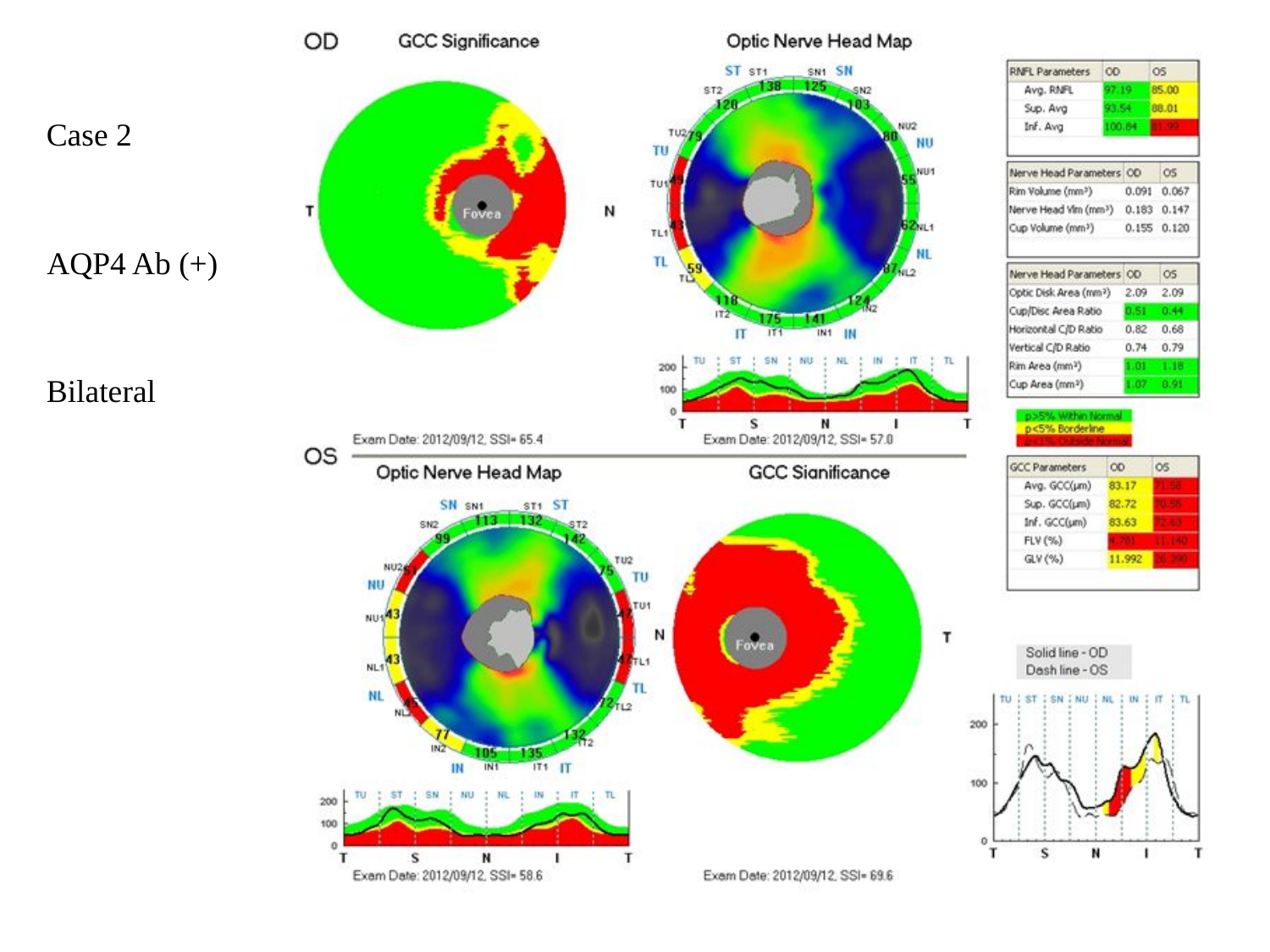

Case 2
AQP4 Ab (+)
Bilateral

## Slide 3
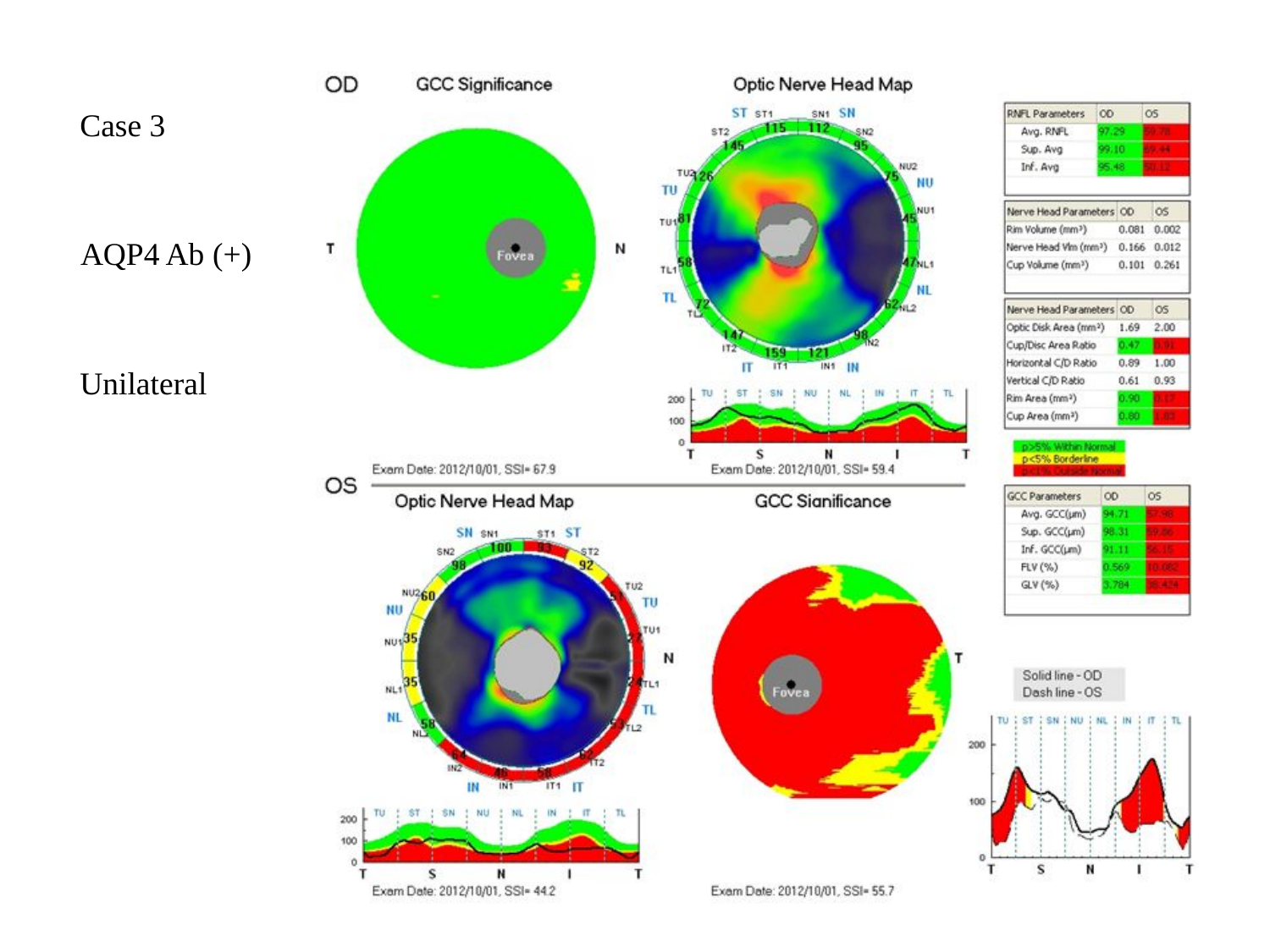

Case 3
AQP4 Ab (+)
Unilateral

## Slide 4
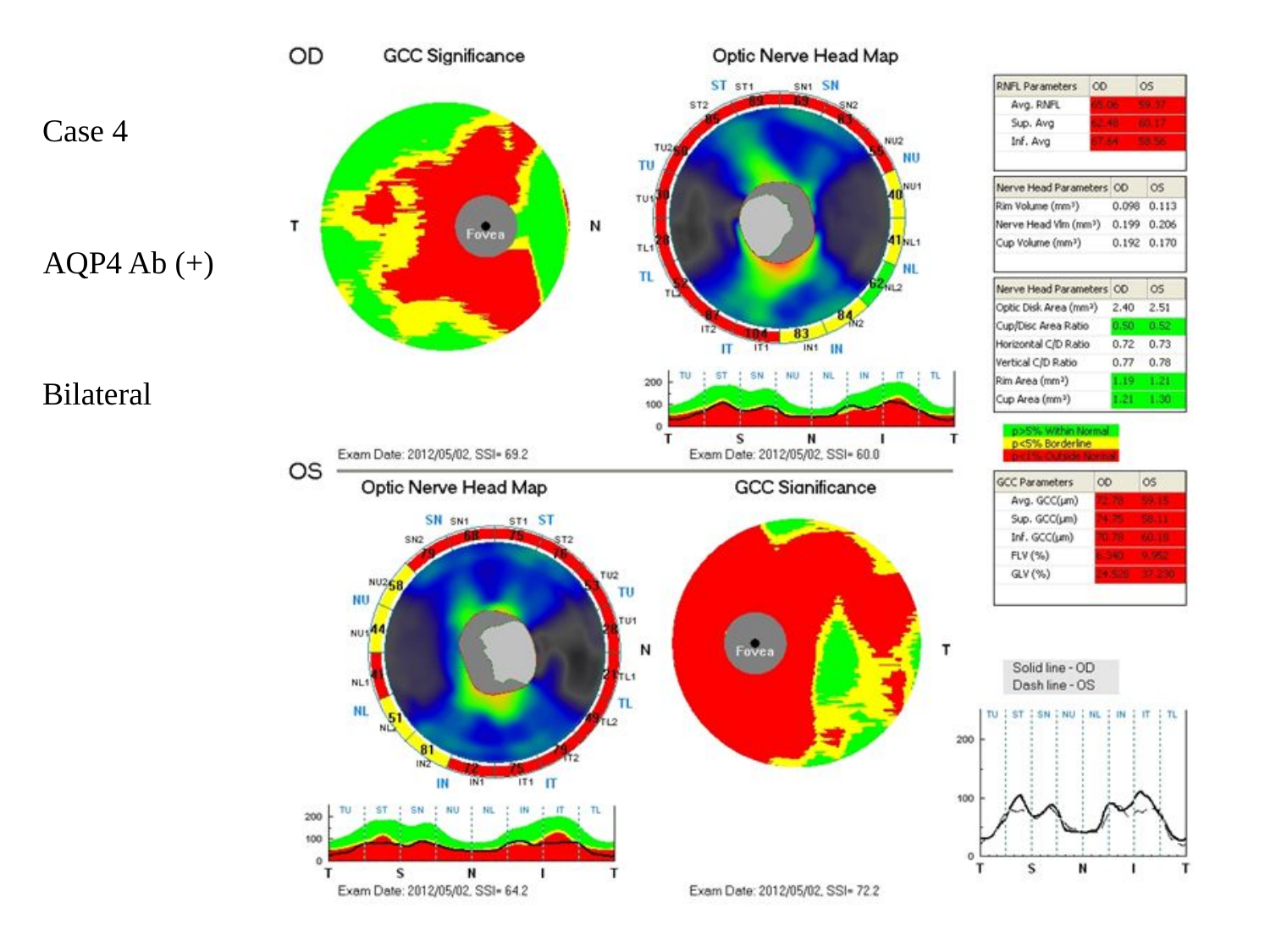

Case 4
AQP4 Ab (+)
Bilateral

## Slide 5
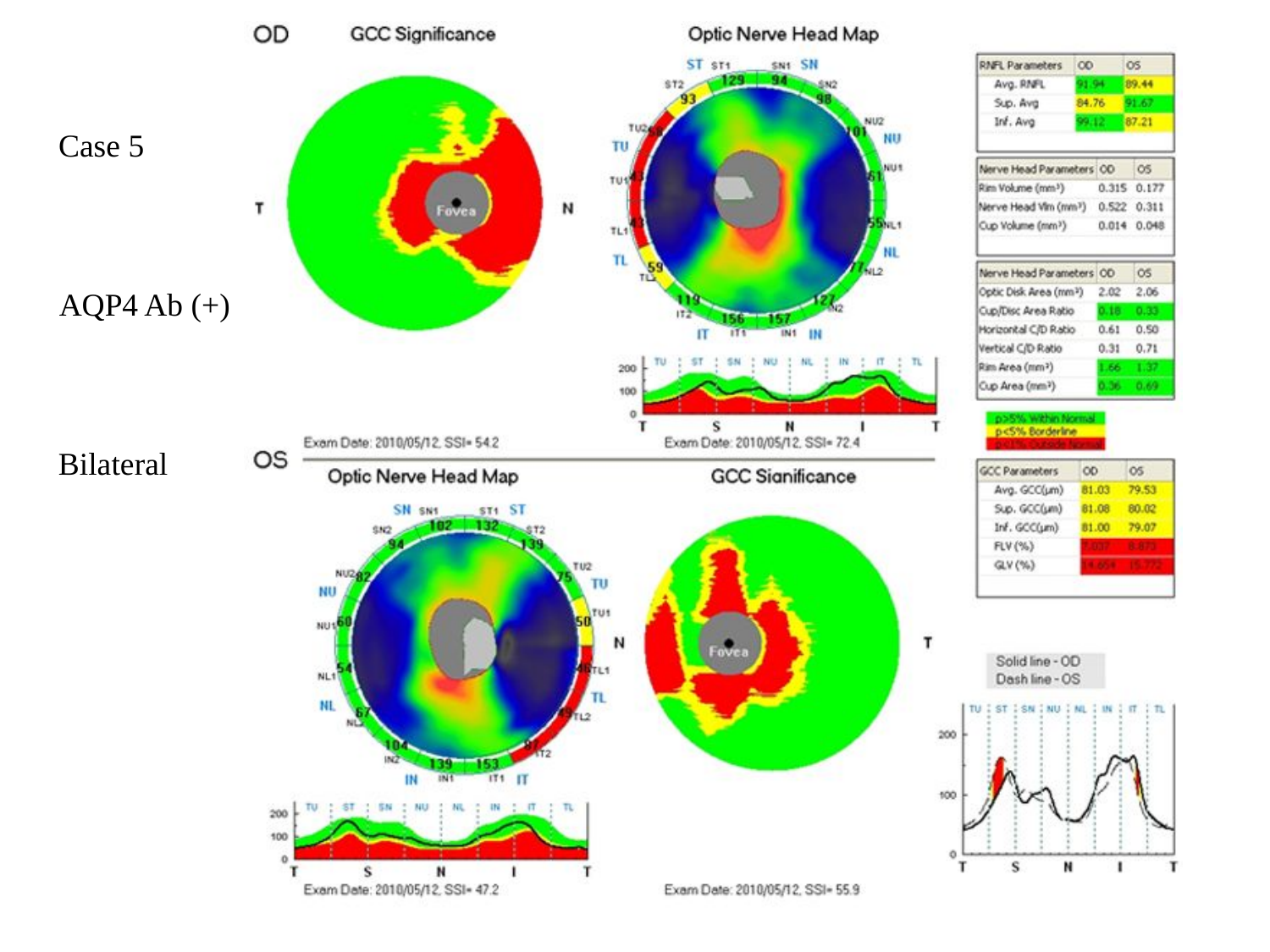

Case 5
AQP4 Ab (+)
Bilateral

## Slide 6
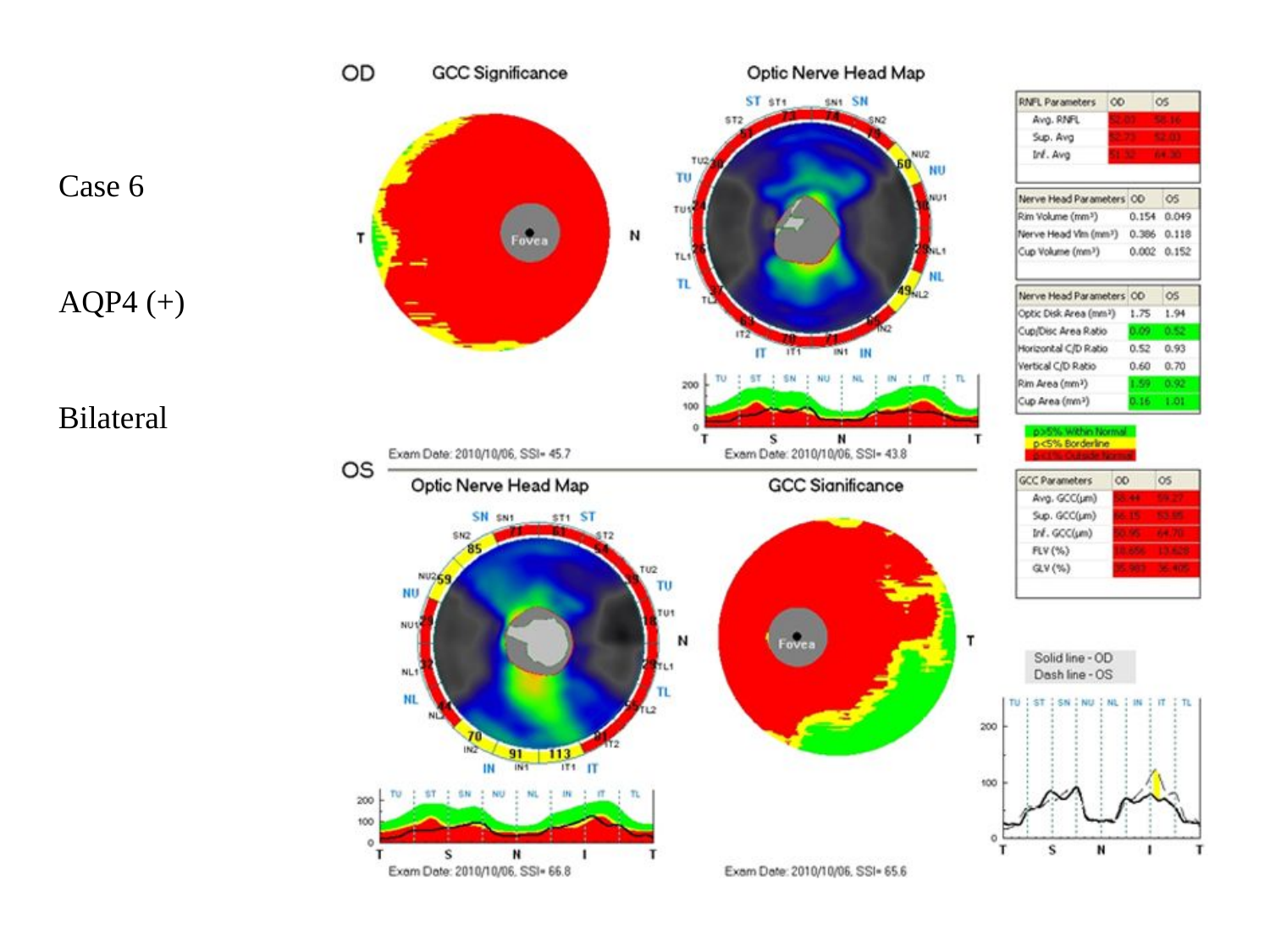

Case 6
AQP4 (+)
Bilateral

## Slide 7
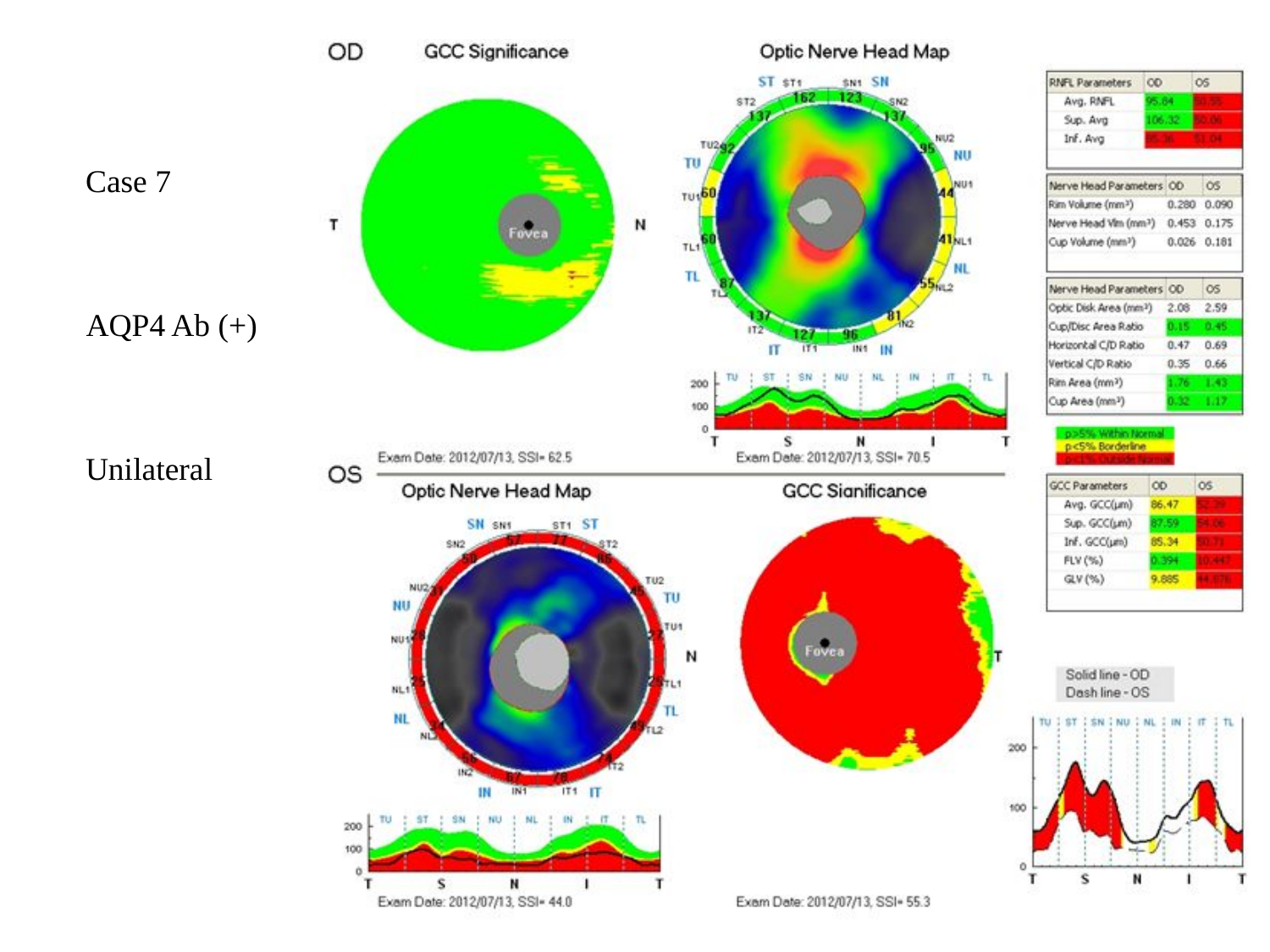

Case 7
AQP4 Ab (+)
Unilateral

## Slide 8
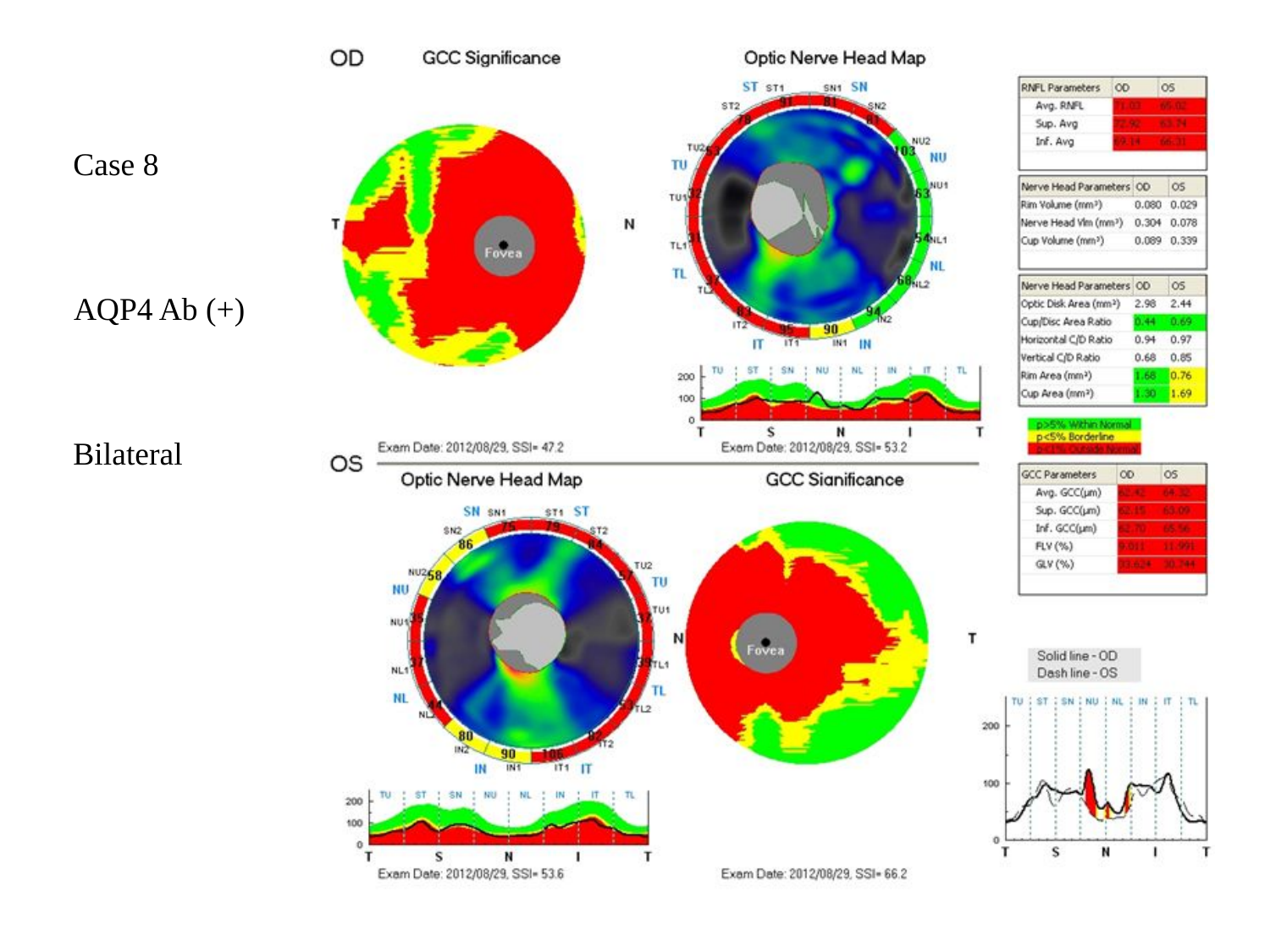

Case 8
AQP4 Ab (+)
Bilateral

## Slide 9
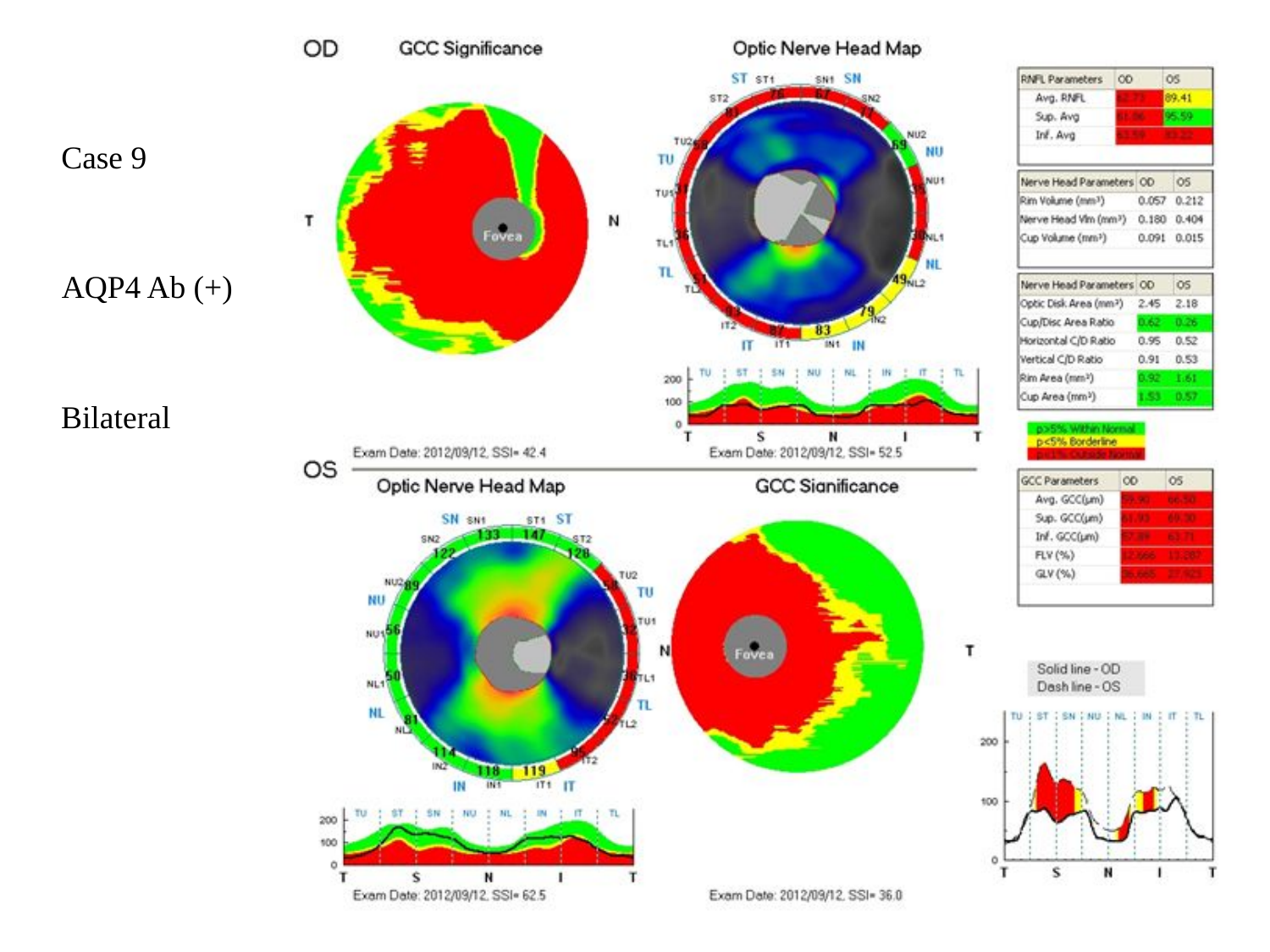

Case 9
AQP4 Ab (+)
Bilateral

## Slide 10
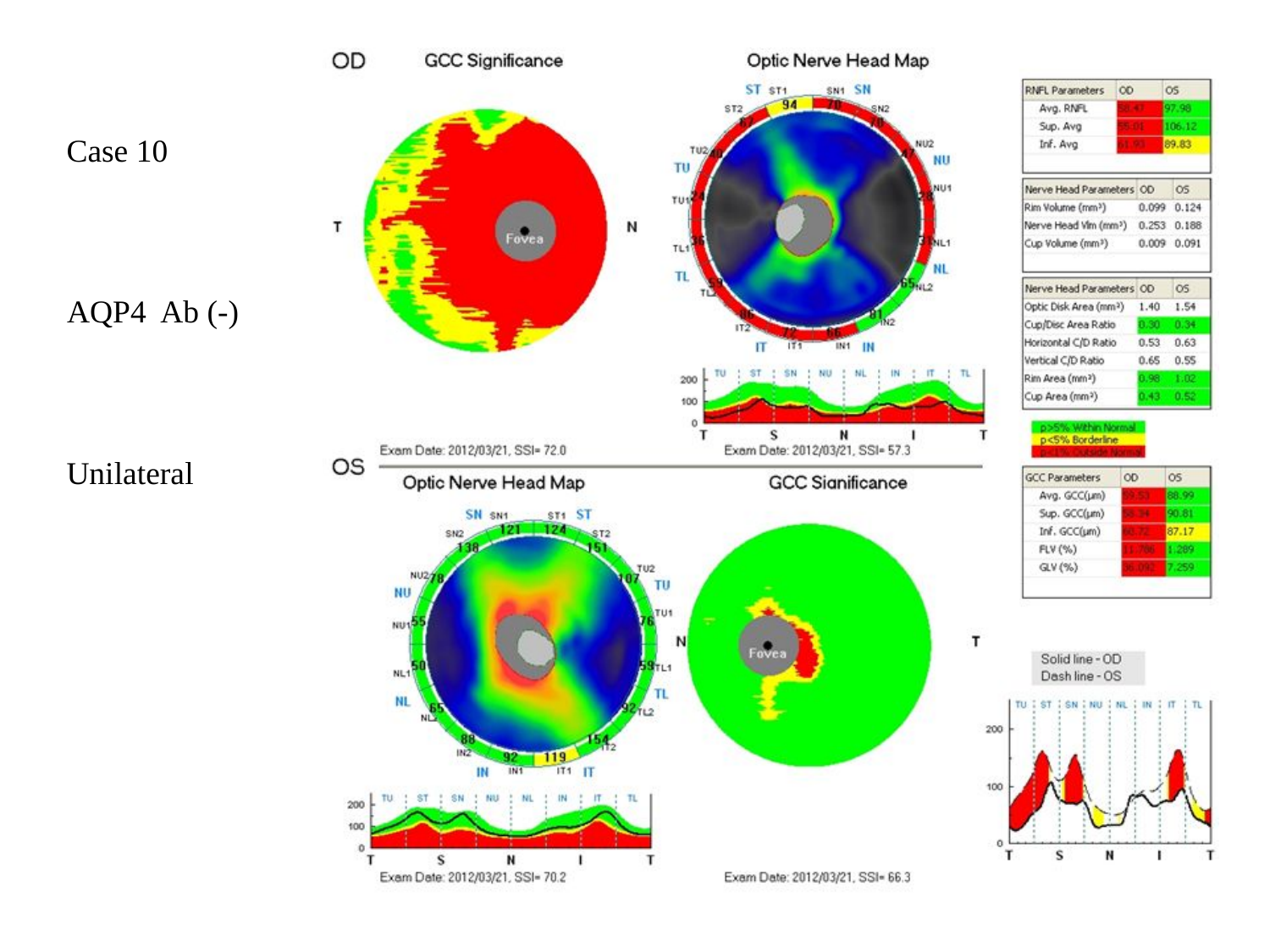

Case 10
AQP4 Ab (-)
Unilateral

## Slide 11
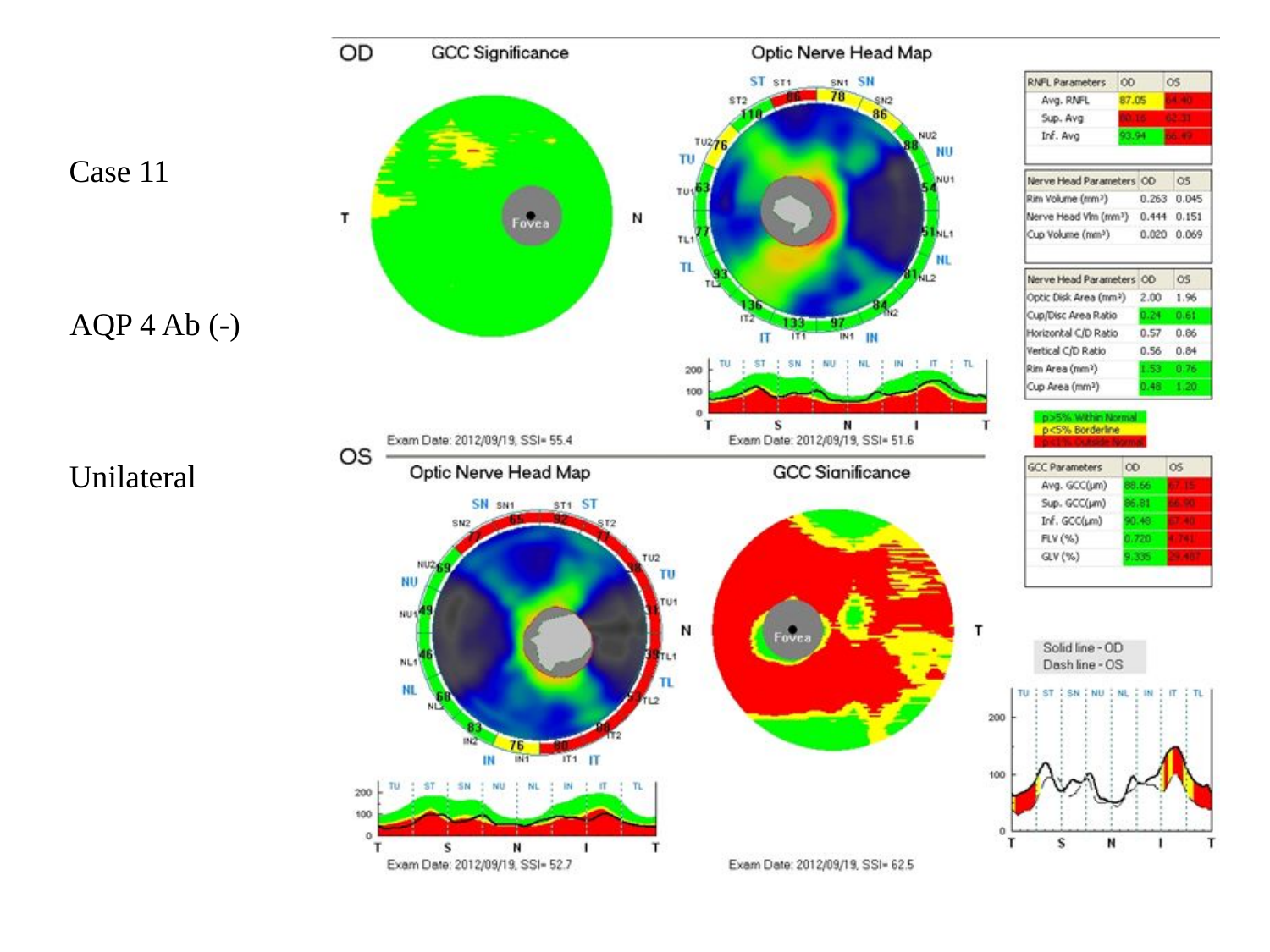

Case 11
AQP 4 Ab (-)
Unilateral

## Slide 12
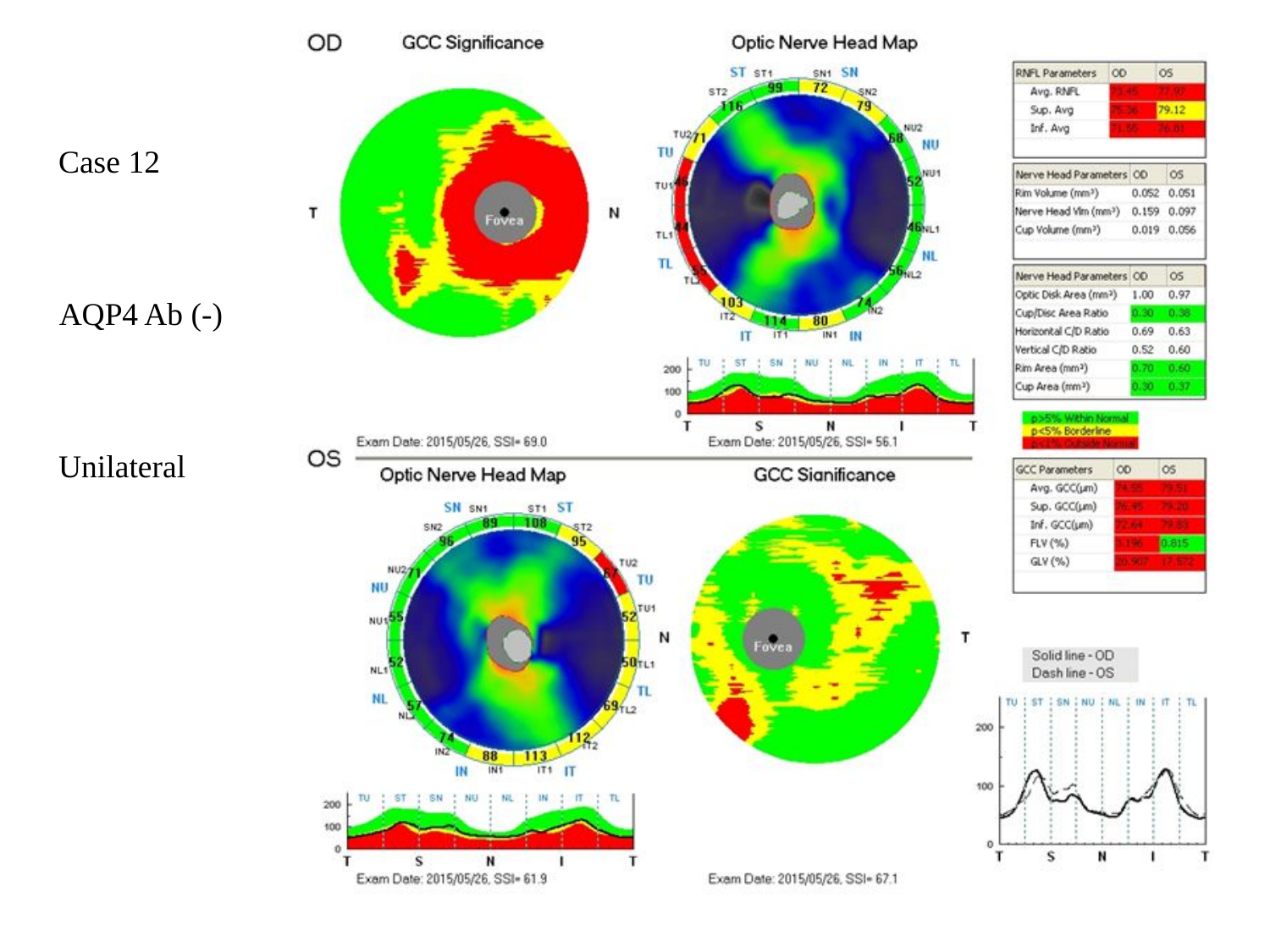

Case 12
AQP4 Ab (-)
Unilateral

## Slide 13
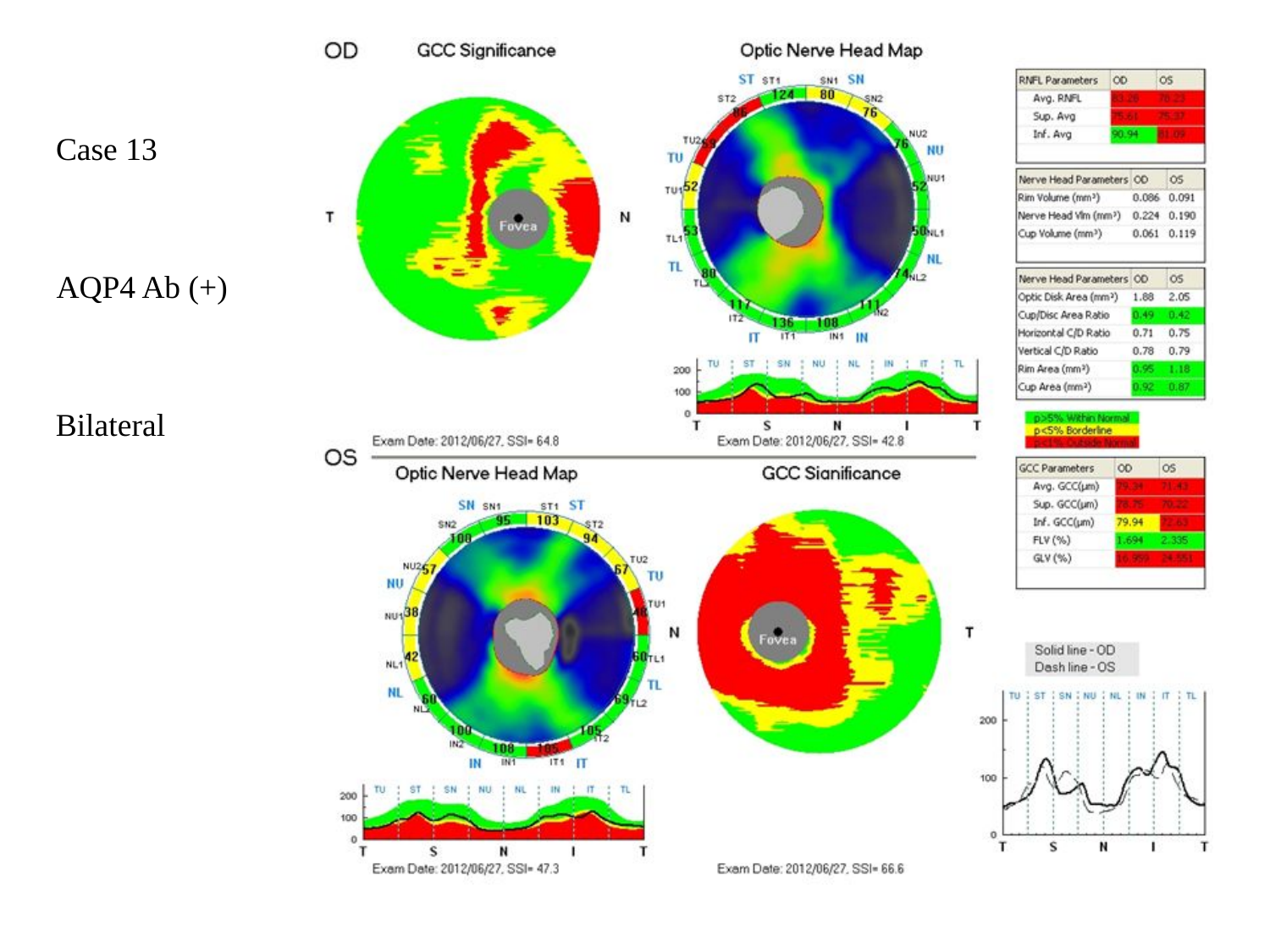

Case 13
AQP4 Ab (+)
Bilateral

## Slide 14
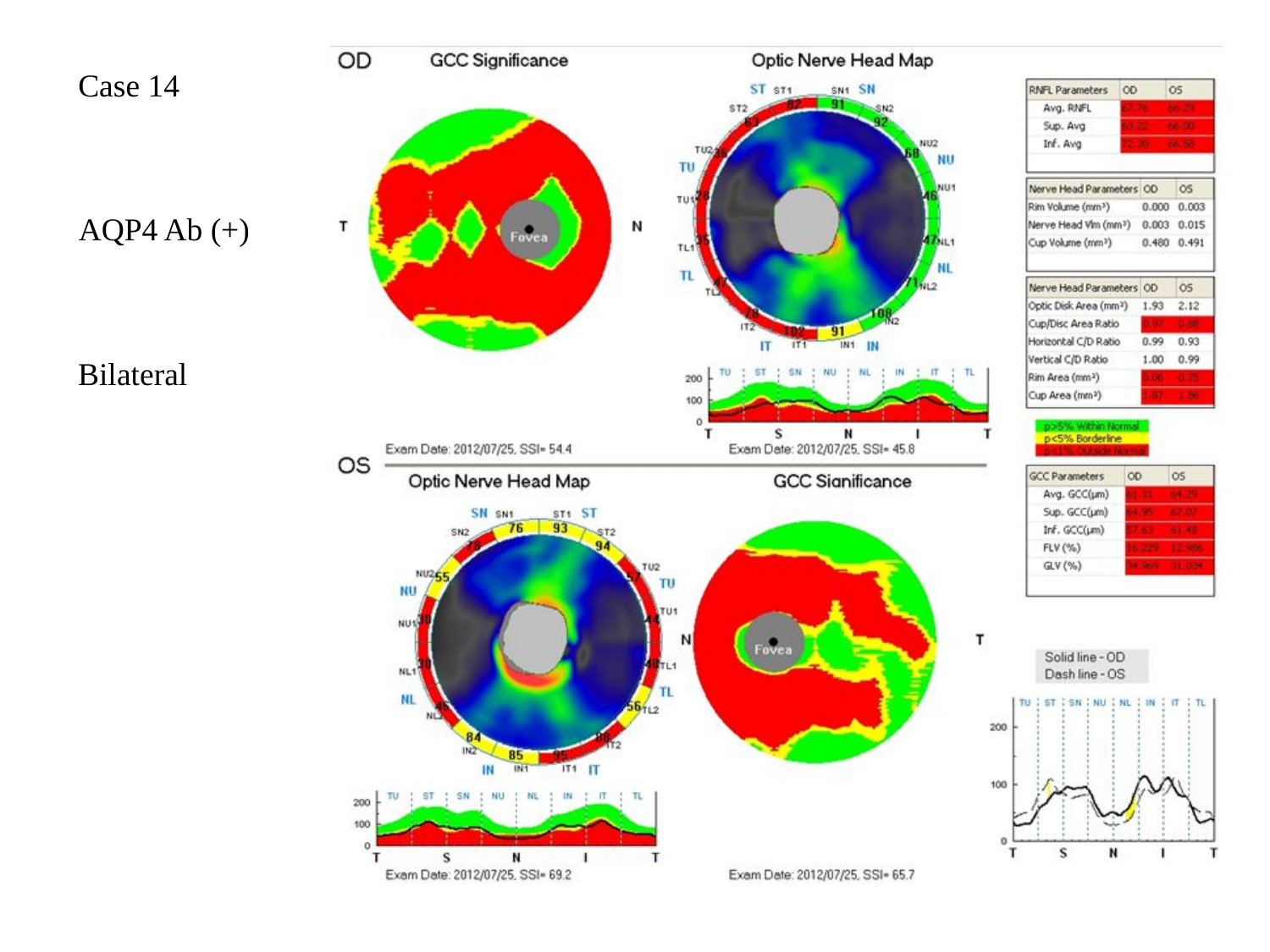

Case 14
AQP4 Ab (+)
Bilateral

## Slide 15
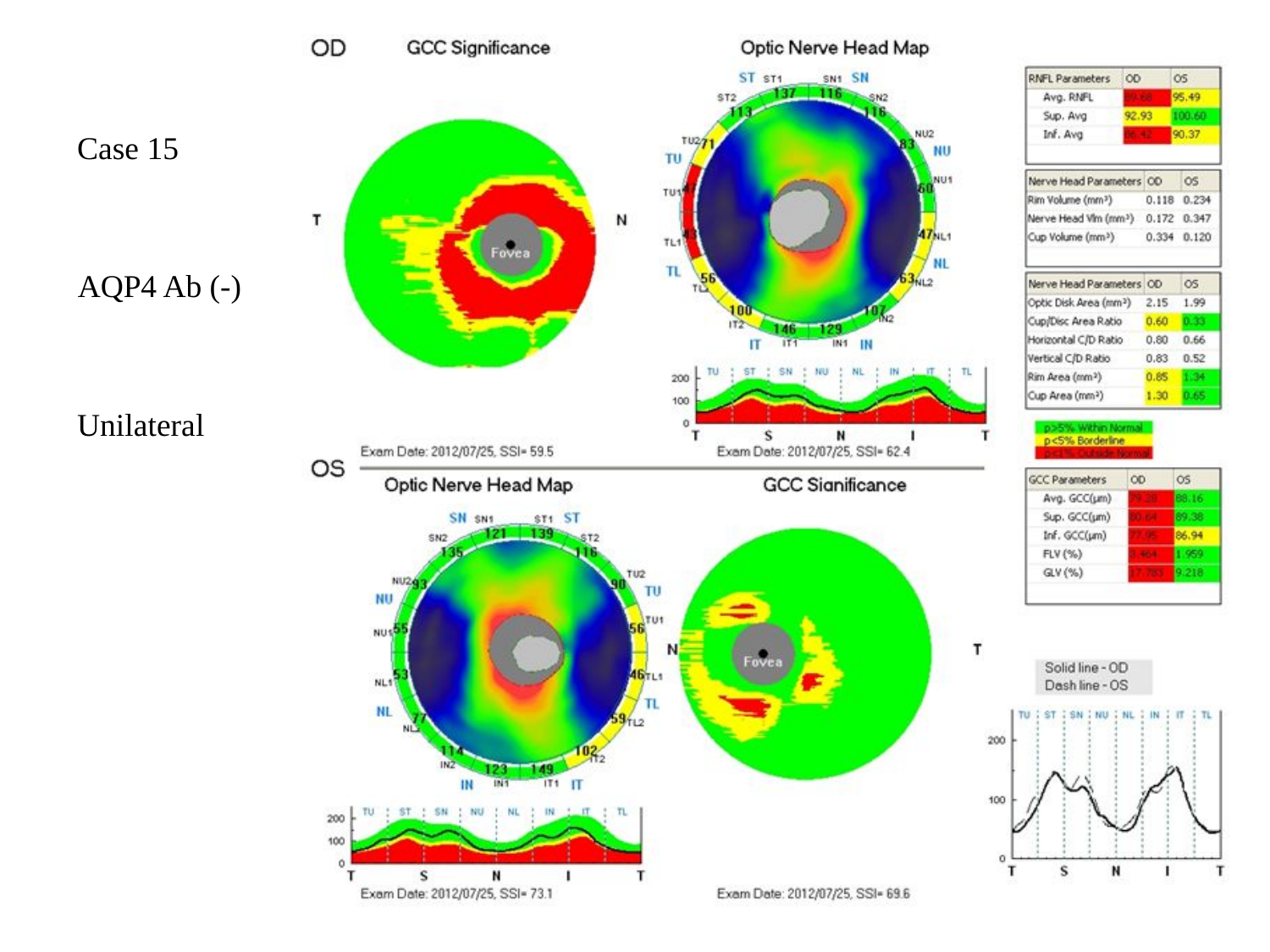

Case 15
AQP4 Ab (-)
Unilateral

## Slide 16
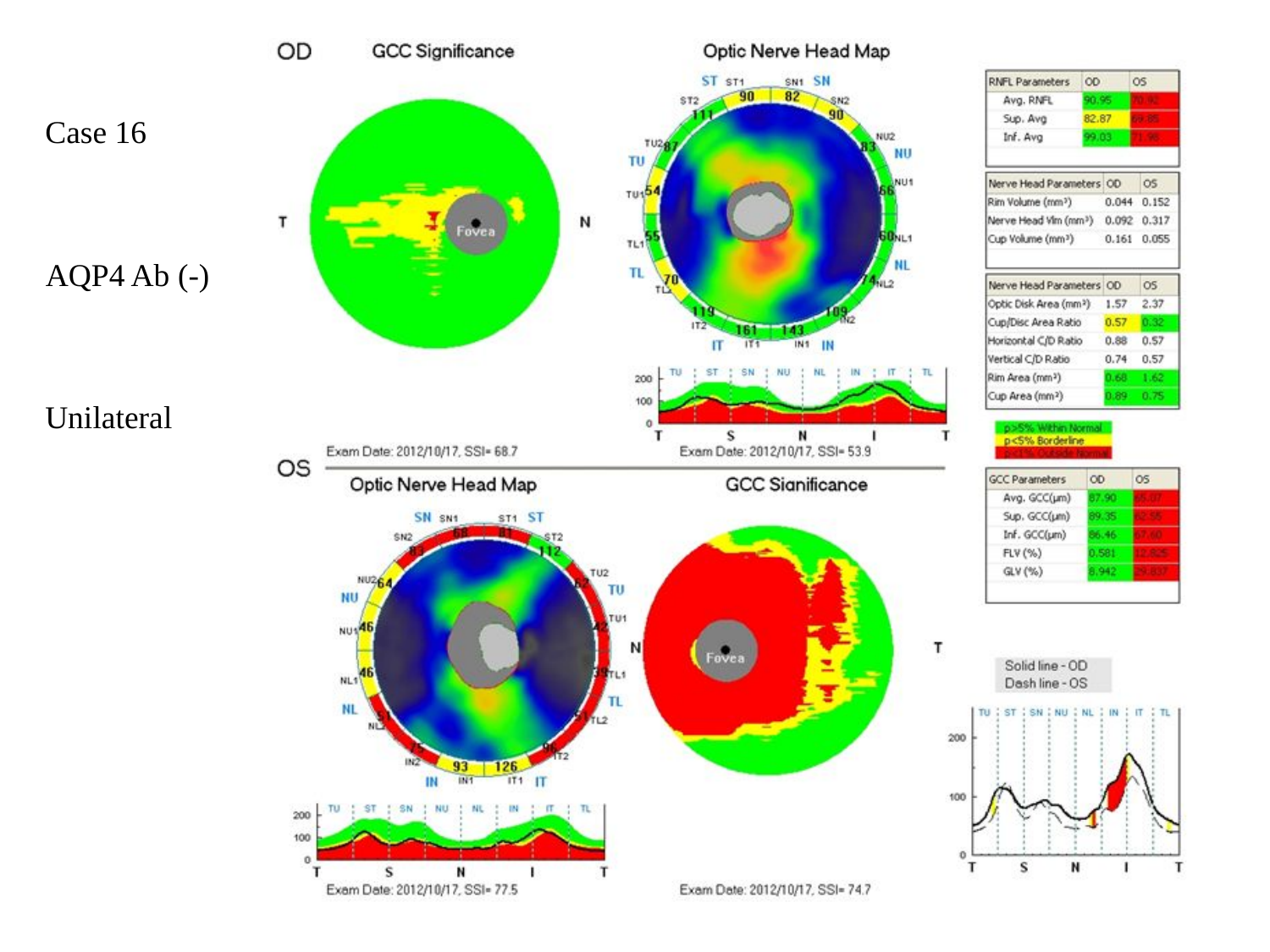

Case 16
AQP4 Ab (-)
Unilateral

## Slide 17
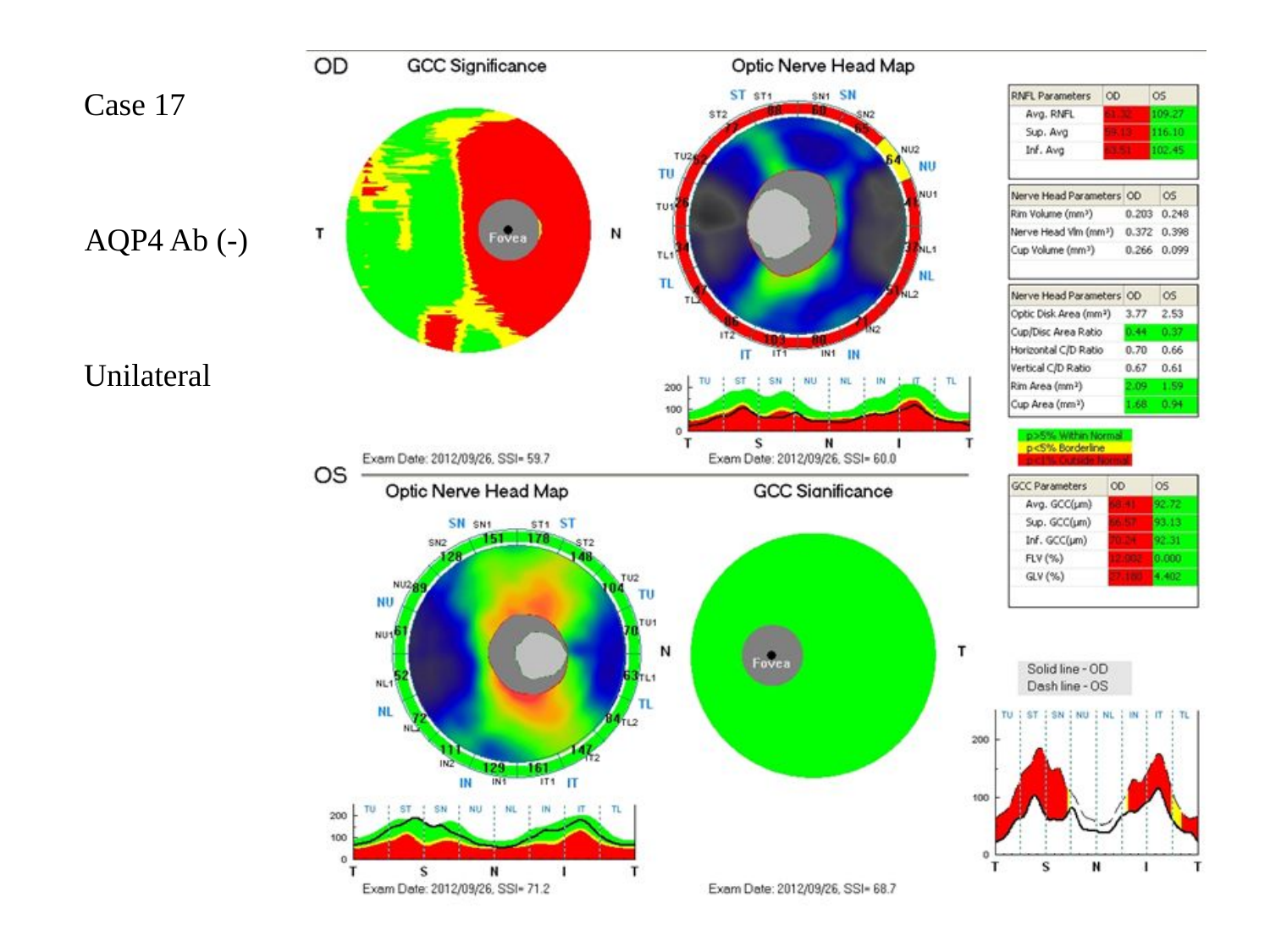

Case 17
AQP4 Ab (-)
Unilateral

## Slide 18
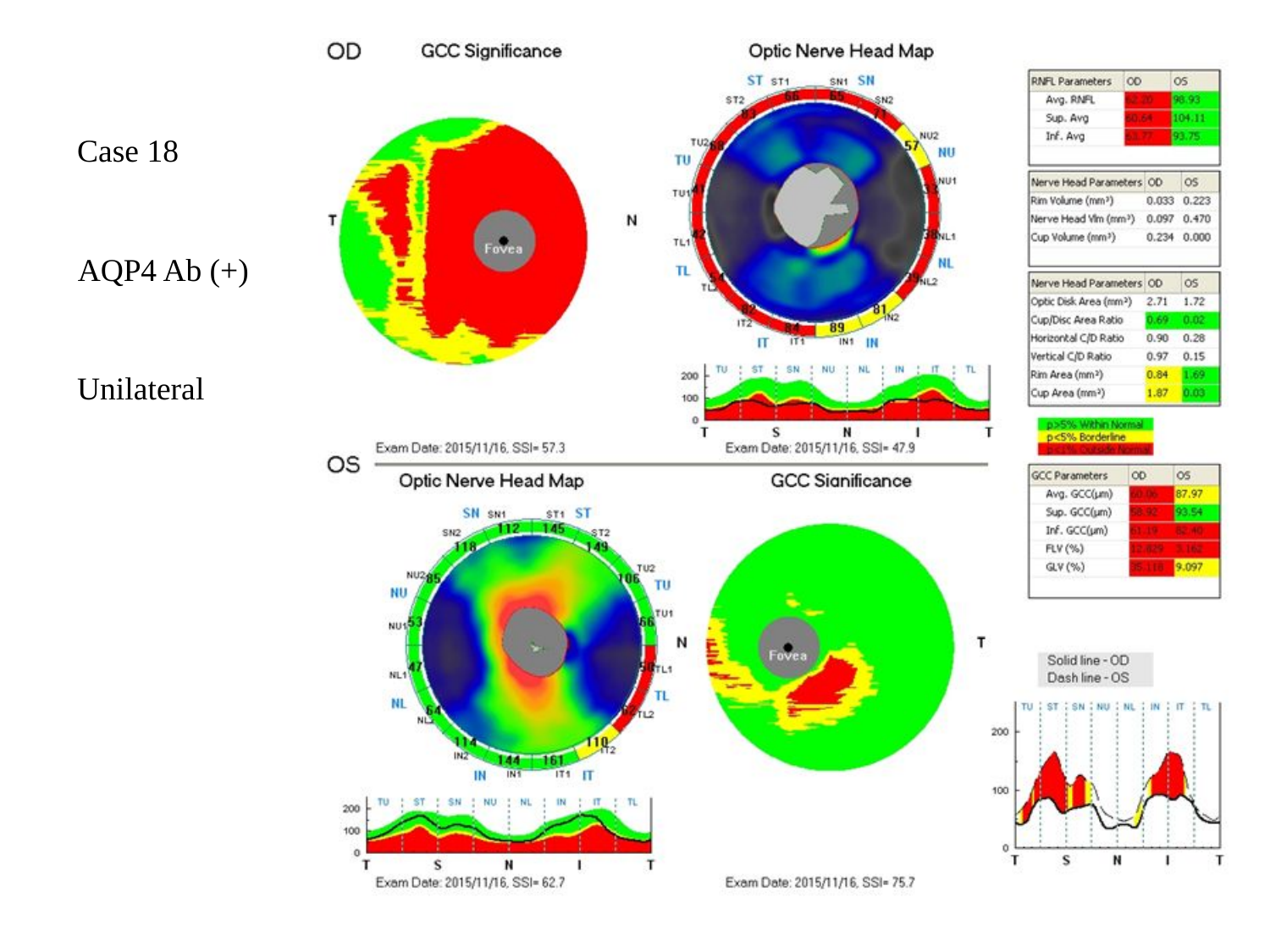

Case 18
AQP4 Ab (+)
Unilateral

## Slide 19
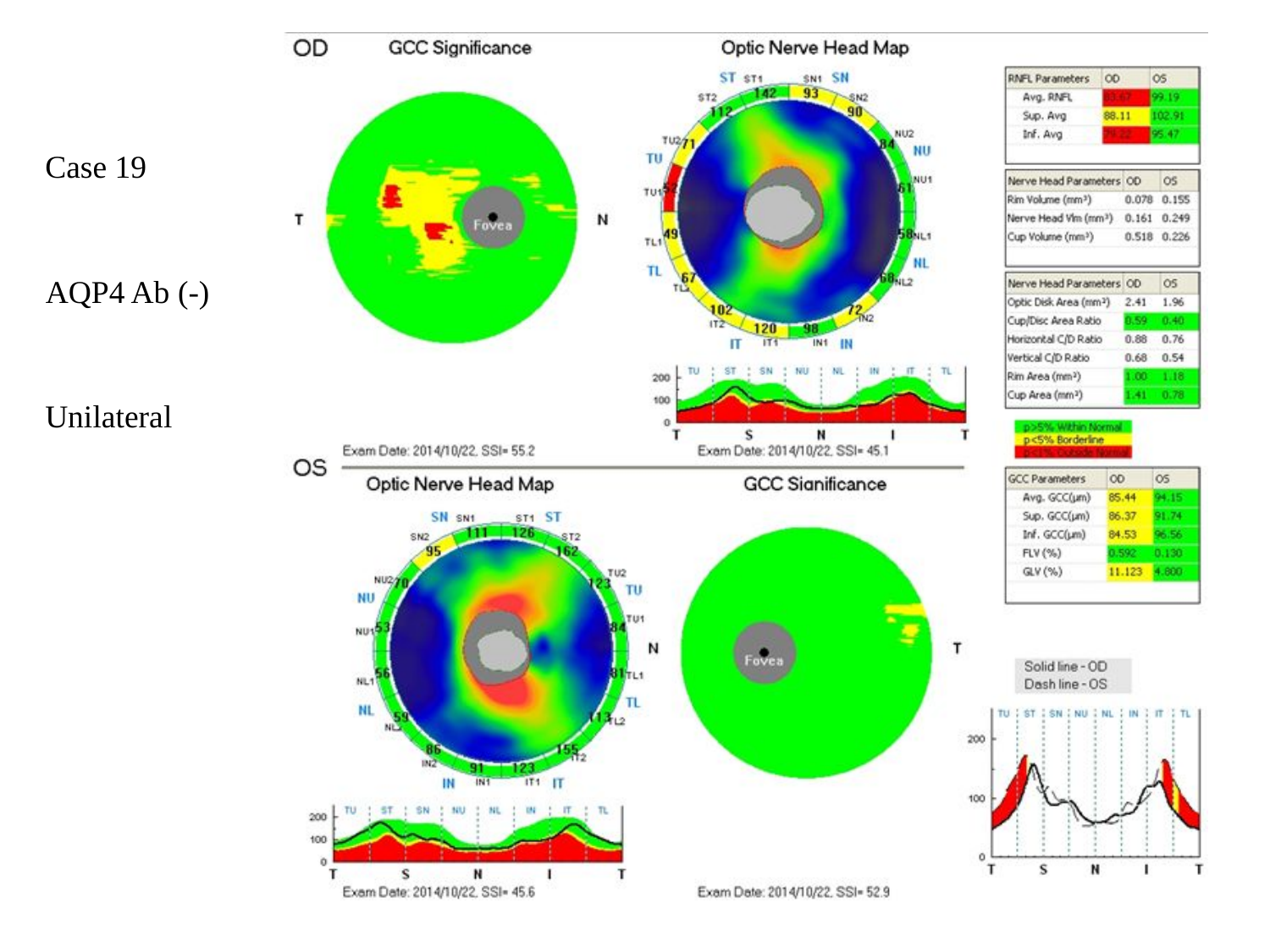

Case 19
AQP4 Ab (-)
Unilateral

## Slide 20
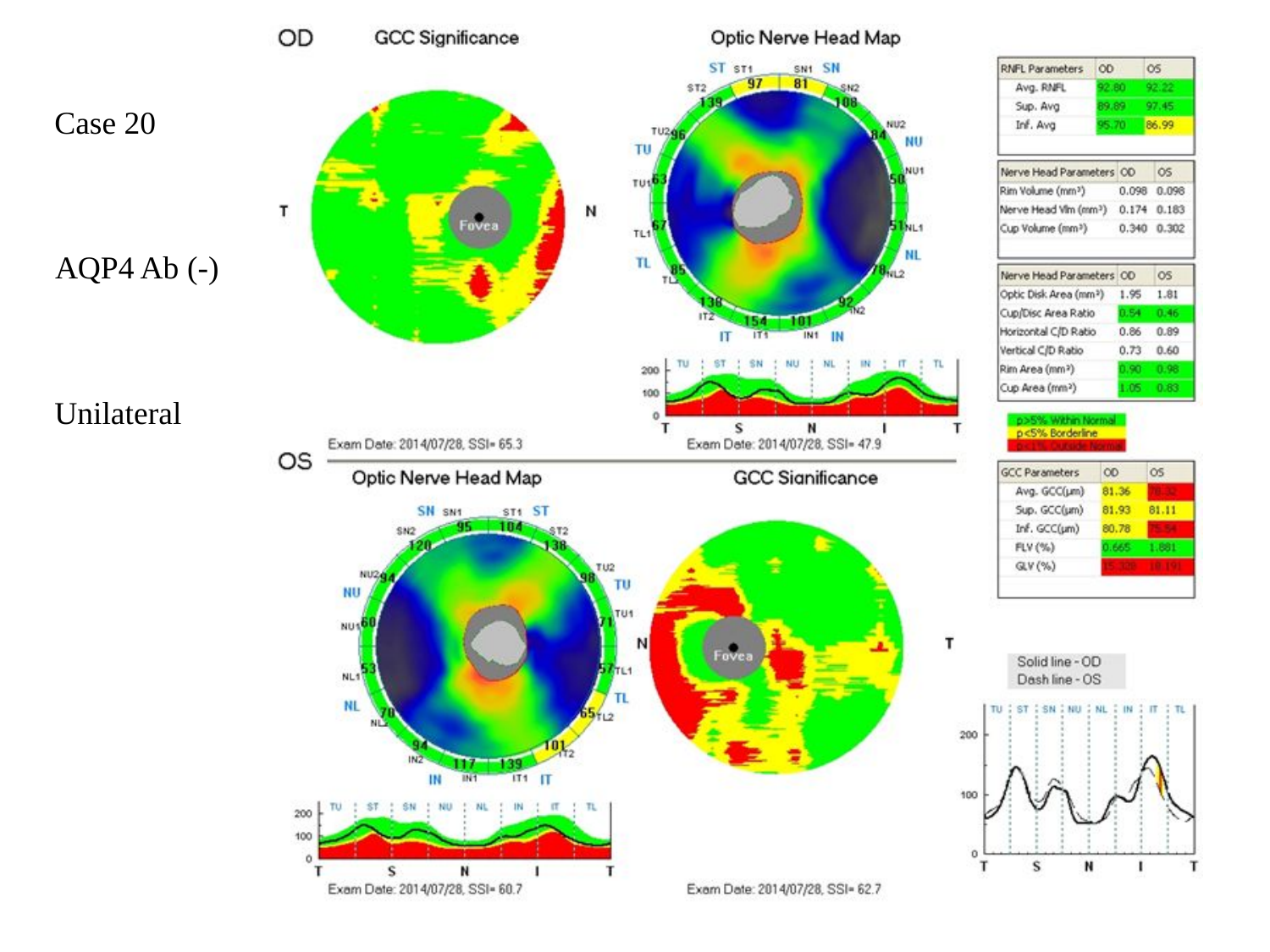

Case 20
AQP4 Ab (-)
Unilateral

## Slide 21
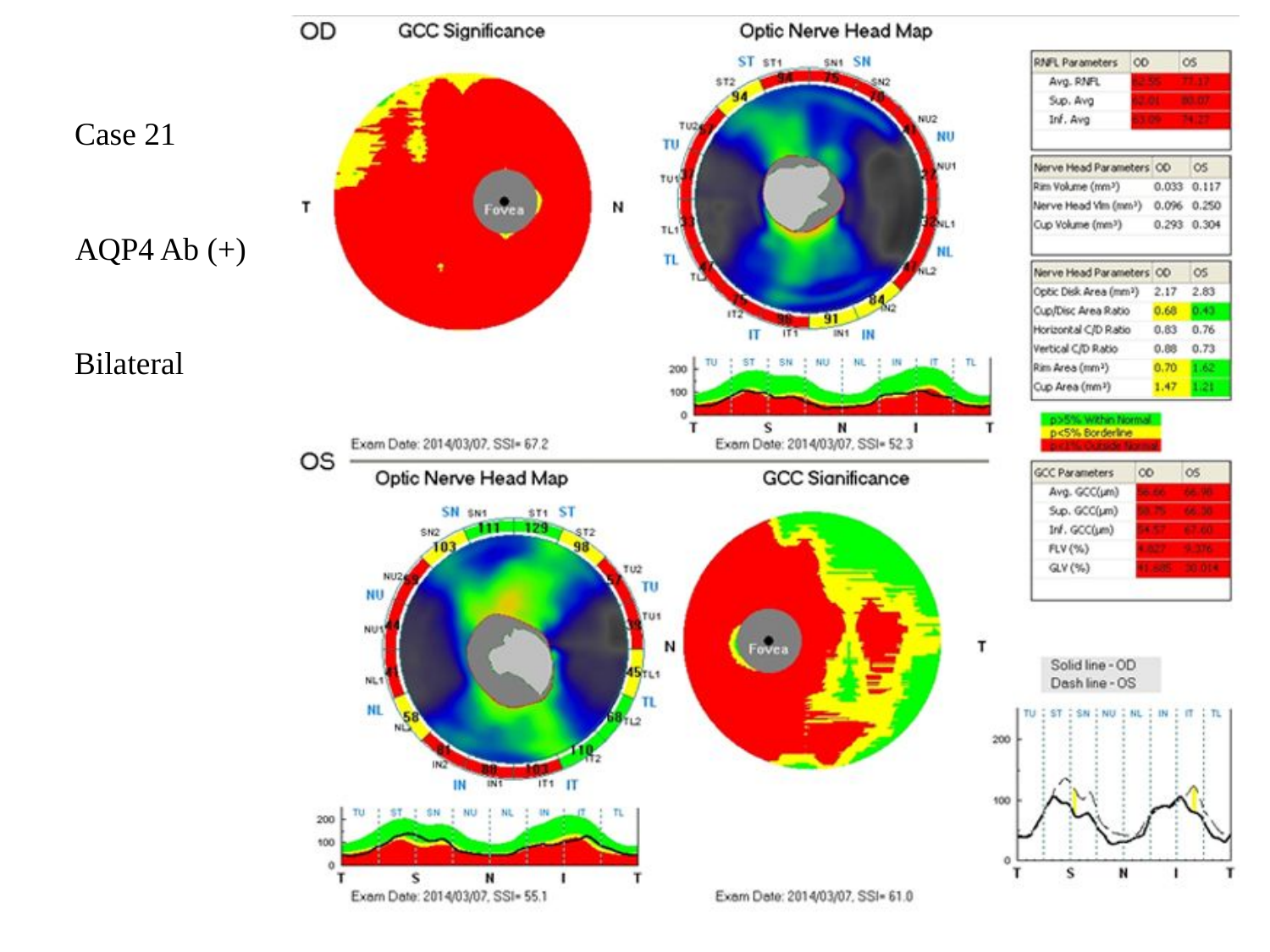

Case 21
AQP4 Ab (+)
Bilateral

## Slide 22
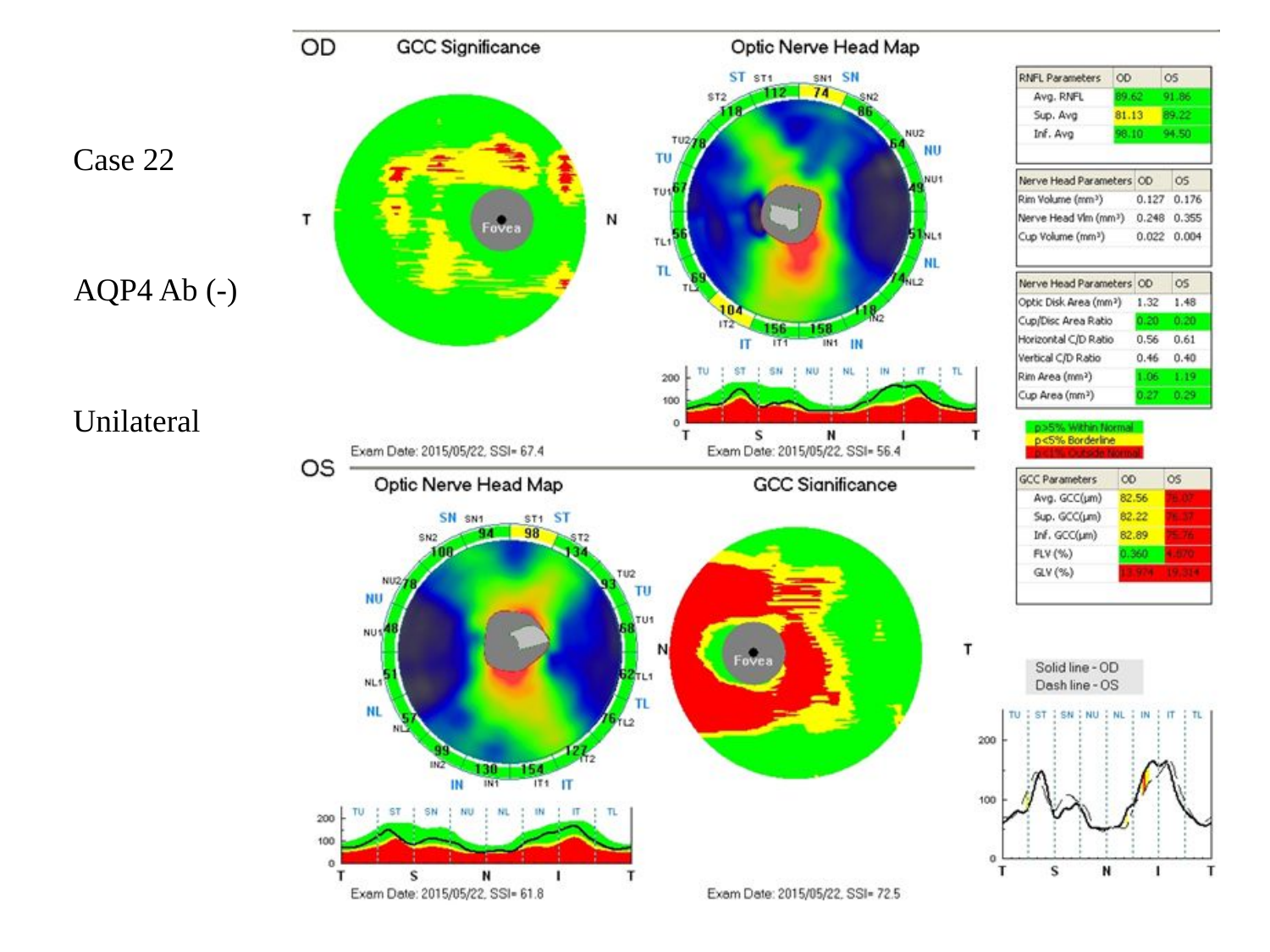

Case 22
AQP4 Ab (-)
Unilateral

## Slide 23
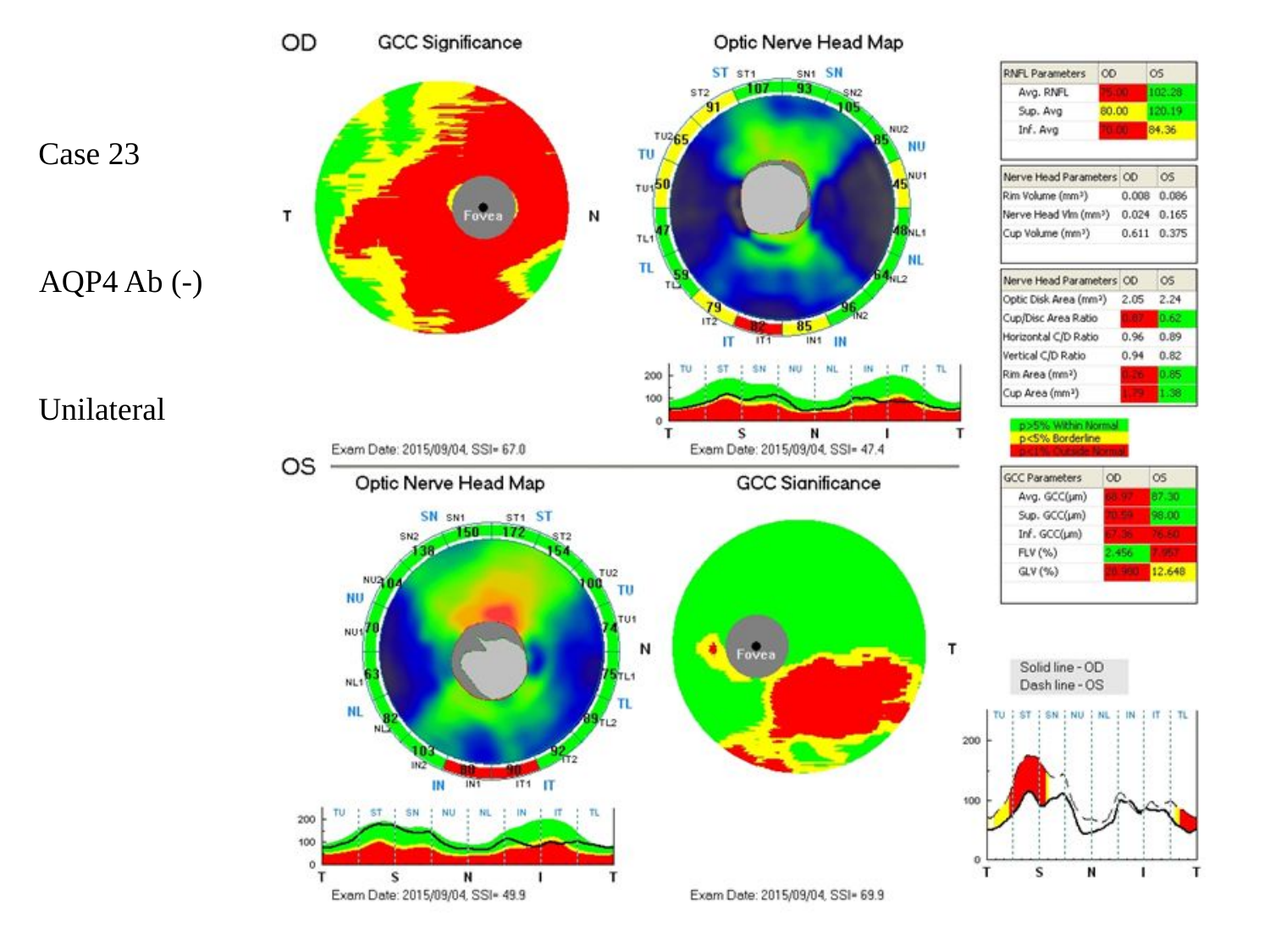

Case 23
AQP4 Ab (-)
Unilateral

## Slide 24
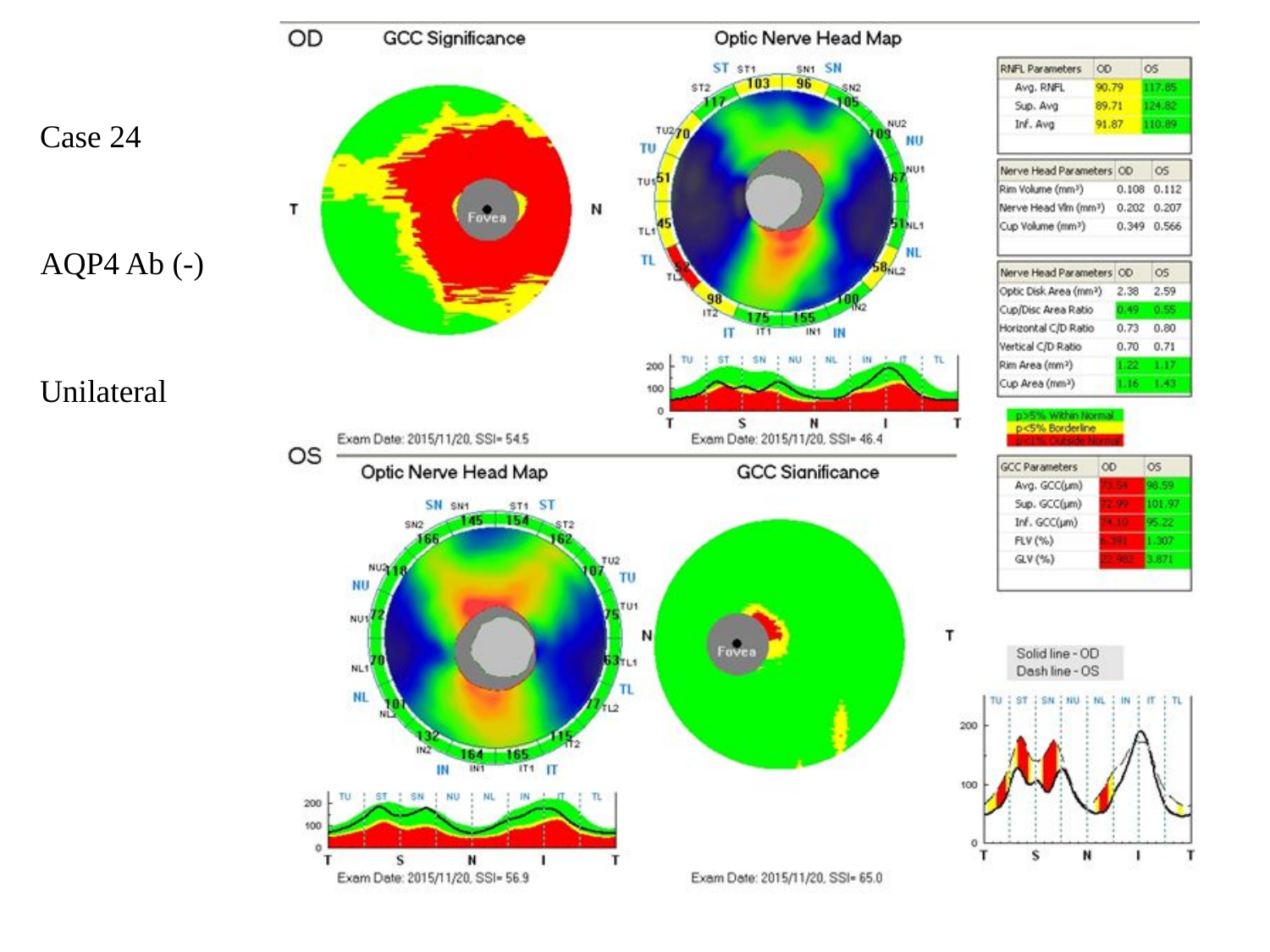

Case 24
AQP4 Ab (-)
Unilateral

## Slide 25
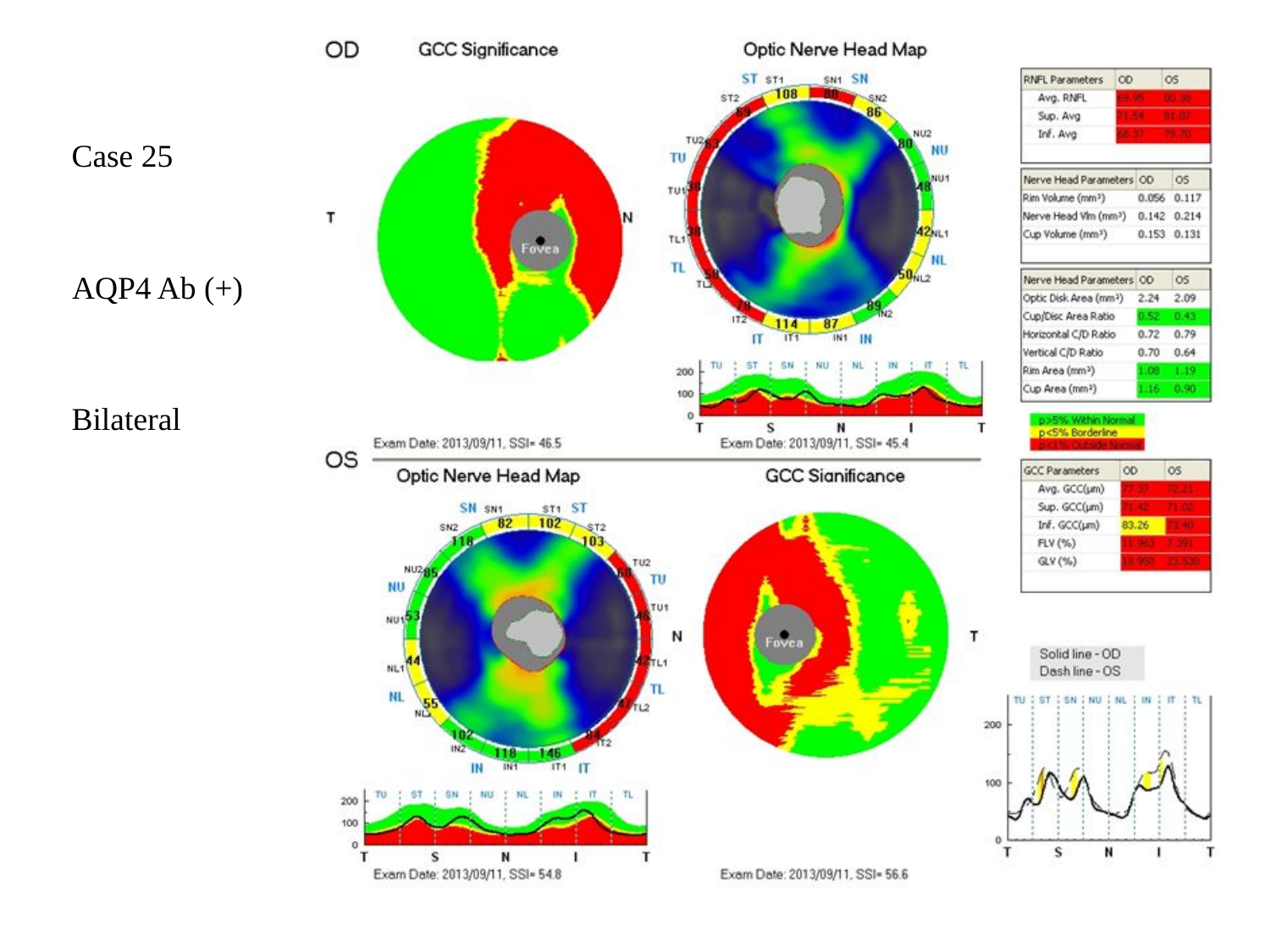

Case 25
AQP4 Ab (+)
Bilateral
